# Use of earth observation-derived hydrometeorological variables to model and predict rotavirus infection (MAL-ED): a multisite cohort study

**DOI:** 10.1016/S2542-5196(19)30084-1

**Published:** 2019-06

**Authors:** Josh M Colston, Benjamin Zaitchik, Gagandeep Kang, Pablo Peñataro Yori, Tahmeed Ahmed, Aldo Lima, Ali Turab, Esto Mduma, Prakash Sunder Shrestha, Pascal Bessong, Roger D Peng, Robert E Black, Lawrence H Moulton, Margaret N Kosek

**Affiliations:** a Department of International Health, Johns Hopkins Bloomberg School of Public Health, Baltimore, MD, USA; b Department of Biostatistics, Johns Hopkins Bloomberg School of Public Health, Baltimore, MD, USA; c Department of Earth and Planetary Sciences, Johns Hopkins Krieger School of Arts and Sciences, Baltimore, MD, USA; d Christian Medical College, Vellore, India; e Nutrition & Clinical Services Division, International Centre for Diarrhoeal Disease Research, Bangladesh (icddr,b), Dhaka, Bangladesh; f Federal University of Ceará, Fortaleza, Brazil; g Interactive Research and Development, Maternal and Child Health (MCH) Program, Karachi, Pakistan; h Haydom Global Health Institute, Haydom, Tanzania; i Department of Child Health, Institute of Medicine of Tribhuvan University, Kathmandu, Nepal; j University of Venda, Thohoyandou, South Africa

## Abstract

**Background:**

Climate change threatens to undermine recent progress in reducing global deaths from diarrhoeal disease in children. However, the scarcity of evidence about how individual environmental factors affect transmission of specific pathogens makes prediction of trends under different climate scenarios challenging. We aimed to model associations between daily estimates of a suite of hydrometeorological variables and rotavirus infection status ascertained through community-based surveillance.

**Methods:**

For this analysis of multisite cohort data, rotavirus infection status was ascertained through community-based surveillance of infants in the eight-site MAL-ED cohort study, and matched by date with earth observation estimates of nine hydrometeorological variables from the Global Land Data Assimilation System: daily total precipitation volume (mm), daily total surface runoff (mm), surface pressure (mbar), wind speed (m/s), relative humidity (%), soil moisture (%), solar radiation (W/m^2^), specific humidity (kg/kg), and average daily temperatures (°C). Lag relationships, independent effects, and interactions were characterised by use of modified Poisson models and compared with and without adjustment for seasonality and between-site variation. Final models were created with stepwise selection of main effects and interactions and their validity assessed by excluding each site in turn and calculating Tjur's Coefficients of Determination.

**Findings:**

All nine hydrometeorological variables were significantly associated with rotavirus infection after adjusting for seasonality and between-site variation over multiple consecutive or non-consecutive lags, showing complex, often non-linear associations that differed by symptom status and showed considerable mutual interaction. The final models explained 5·9% to 6·2% of the variability in rotavirus infection in the pooled data and their predictions explained between 0·0% and 14·1% of the variability at individual study sites.

**Interpretation:**

These results suggest that the effect of climate on rotavirus transmission was mediated by four independent mechanisms: waterborne dispersal, airborne dispersal, virus survival on soil and surfaces, and host factors. Earth observation data products available at a global scale and at subdaily resolution can be combined with longitudinal surveillance data to test hypotheses about routes and drivers of transmission but showed little potential for making predictions in this setting.

**Funding:**

Bill & Melinda Gates Foundation; Foundation for the National Institutes of Health, National Institutes of Health, Fogarty International Center; Sherrilyn and Ken Fisher Center for Environmental Infectious Diseases, Johns Hopkins School of Medicine; and NASA's Group on Earth Observations Work Programme.

## Introduction

The global burden of diarrhoeal disease, the fourth leading cause of under-5 mortality, is likely to increase because of climate change. In 2030 there will be an estimated 48 000 additional deaths from diarrhoea, brought about by shifts in environmental and meteorological factors influencing transmission of pathogens that cause enteric infectious diseases.^1^, ^2^, ^3^ These climate-related shifts threaten to undermine recent progress in reducing childhood deaths from diarrhoeal disease, but the absence of evidence about how individual environmental factors affect transmission of specific pathogens makes prediction of trends under particular climate scenarios challenging.^3^ Recognising the complexity of the ecological systems within which weather influences enteric infectious diseases, there have been calls for further research to characterise the associations between multiple meteorological exposures and individual infectious agents that cause enteric infectious diseases.^4^, ^5^

Rotavirus, the pathogen responsible for the largest share of the vaccine-preventable diarrhoeal disease burden, is perhaps the enteric infectious disease for which relationships with climate have been best characterised.^6^ Associations between several meteorological exposures—notably, temperature, humidity, and precipitation^7^, ^8^, ^9^—and rotavirus outcomes have been documented at various locations. Three hypothesised pathways are discussed below.

**Research in context**

**Evidence before this study**

Changes in the distribution and burden of diarrhoeal diseases are one of the principal manifestations by which climate change is predicted to affect human health, as was confirmed by an exploratory review of the literature. Rotavirus is the enteric pathogen for which associations with climate factors have been most thoroughly explored. Numerous studies and reviews have attempted to characterise associations between meteorological exposures and either general diarrhoeal disease or rotavirus-specific outcomes at particular locations, with several reporting relationships with temperature, relative humidity, and precipitation. Associations with river levels, atmospheric pressure, wind speed, and solar radiation have also been documented. Hypothesised explanations for these findings emphasise three possible pathways: survivability of the virus outside the host, dispersion of the virus through the environment, and host behavioural factors. However, several questions remain about the relative importance of these mechanisms.

**Added value of this study**

This study incorporates information from eight different epidemiological surveillance sites representing different climate zones and is, to our knowledge, the first to combine multiple climate variables, single-day hydrometeorological exposure estimates, differential lag relationships, interactions, non-linearity, and adjustment for seasonality. It also assesses for the first time the validity of models based on climate data in predicting site-specific rotavirus disease burden.

**Implications of all the available evidence**

Numerous hydrometeorological variables show complex, non-linear associations with rotavirus infection that differ by episode type and operate over different lag lengths, including as short a period as 2 days. Available evidence suggests that the effect of climate on rotavirus transmission is mediated by four independent mechanisms: waterborne dispersal, airborne dispersal, virus survival on soil and surfaces, and host factors. Changes in weather patterns might alter the rotavirus disease burden in different directions depending on the climate zone and whether the rotavirus vaccine has been introduced.

The first is survival of the virus outside the host: atmospheric and soil conditions might affect the duration of survival of rotavirus when suspended in airborne aerosols or adhered to surfaces and fomites.^8^, ^10^ The second is dispersal of the virus through the environment: rainfall runoff is a means by which viruses disseminate and collect in surface water or groundwater or are rinsed from the soil,^4^ while wind and pressure might promote their aerial transport.^5^ The third is host factors and behaviours: human responses to weather conditions might mediate or interact with climate exposures. For example, rainfall promotes contact between infected and susceptible individuals as they congregate indoors and seasonal changes in diet or water sources alter exposure to pathogens.^11^

Questions remain about the relative importance of these pathways, and there is a need for an approach that can be applied systematically to evaluate the combined impact of multiple meteorological exposures to pathogen-specific enteric infectious disease outcomes at a level of spatiotemporal disaggregation sufficient to characterise potential lag effects, interactions, and non-linearity.^4^, ^5^ Earth observation (EO) climate data products derived from satellites and model-based re-analysis offer an opportunity to address this research gap.^12^

In this analysis of multisite cohort data we aimed to model the associations between daily estimates of nine EO-derived hydrometeorological variables and rotavirus infection status ascertained through community-based surveillance, and to validate the model predictions. The a-priori hypotheses to be tested were that the risk of rotavirus transmission is highest following extremes of precipitation^4^ (very high and very low rainfall), cooler, drier atmospheric conditions,^8^, ^13^, ^14^ increased advective air movement^5^ (which correlates with pressure and wind speed), and lower levels of solar radiation.^8^

## Methods

### Study population

The Etiology Risk Factors and Interactions of Enteric Infections and Malnutrition and the Consequences for Child Health and Development (MAL-ED) project was designed to investigate risk factors for enteric infection, diarrhoeal disease, and undernutrition.^15^ Birth cohorts of 227 to 303 newborn babies were recruited from communities in eight low-income and middle-income countries: Dhaka, Bangladesh; Fortaleza, Brazil; Vellore, India; Bhaktapur, Nepal; Naushero Feroze, Pakistan; Loreto, Peru; Venda, South Africa; and Haydom, Tanzania. Cohorts were monitored continuously over their first 2 years of life from November, 2009, to March, 2014 (the duration of follow-up differed by site). The socioeconomic, health, and environmental contexts of these eight study sites have been described elsewhere.^12^, ^16^, ^17^, ^18^, ^19^, ^20^, ^21^, ^22^, ^23^ Ethical approval for MAL-ED was given by the Johns Hopkins Institutional Review Board as well as from the respective partner institutions at each site. Written consent was obtained from all participants' caregivers.

### Outcome variables

Stool samples were collected from study participants at monthly intervals following enrolment and following reporting of a diarrhoeal episode by the child's caregiver. Rotavirus infection status was ascertained by two methods. Samples from children who remained in the study for up to 24 months of age were tested for rotavirus (and other enteropathogens) with probe-based quantitative PCR assays on custom-developed TaqMan Array Cards (ThermoFisher, Carlsbad, CA, USA).^24^ Samples from participants who did not complete follow-up were assessed for rotavirus positivity by the ProSpecT ELISA diagnostic test (Oxoid, Ely, UK).^25^ Infection status was ascertained for more than 50 000 stool samples from 2100 study participants. Because the date of their collection was recorded, it was possible to match these results with daily meteorological exposure estimates from historical weather data re-analysis by precise date. Enrolment of participants was staggered throughout the calendar year to eliminate effects of age on seasonality, so the resulting data set constituted a continuous time series of rotavirus-positive and rotavirus-negative samples spanning the follow-up period (appendix p 1).

### Exposure variables

The main exposure variables in this analysis were a set of historical daily EO-based and model-based re-analysis-derived estimates of hydrometeorological variables at each MAL-ED study site location drawn from version 2.1 of the Global Land Data Assimilation System (GLDAS).^26^ These data have been described, evaluated, made publicly available, and justified for use in cohort data analyses; their advantages and limitations have been discussed elsewhere.^12^ A script was run to extract all variable values from the gridded GLDAS files (which have a horizontal resolution of 0·25 decimal degrees) for the 2007–16 period at the coordinates of the eight MAL-ED site locations. The 3-hourly estimates were aggregated to daily averages, totals, or minimum and maximum daily values as appropriate. Nine variables were selected on the basis of their hypothesised potential to influence rotavirus transmission via two mechanisms, described below.

The first mechanism was virus dispersal. Waterborne virus dispersal was measured by the daily total precipitation volume (mm) and daily total surface runoff (mm). Airborne virus dispersal was measured by surface pressure (mbar) and wind speed (m/s).

The second mechanism was virus survival, which was measured by relative humidity (%), soil moisture (%), solar radiation (W/m^2^), specific humidity (kg/kg), and average daily temperatures (°C; calculated from the daily minimum and maximum).

### Statistical analysis

The probability of a stool sample being positive for rotavirus on a given day was calculated from risk ratios by fitting modified Poisson models with clustered robust variance estimation to the binary infection status outcome by use of generalised linear models.^27^ Two separate effects of the hydrometeorological exposure variables on the rotavirus outcome were modelled: the absolute effect and the adjusted effect.

Absolute effect analyses made no adjustment for seasonality and therefore incorporated the effect of medium-term, intra-annual variability due to shifts in both climate and rotavirus incidence over the yearly cycle.

For adjusted effect analyses, the hydrometeorological variables were first standardised to their local distributions by recalculating each one as the deviation from its site-specific 10-year mean value (2007–16) to adjust for confounding by between-site variation or by sites that might be outliers with respect to both exposures and outcome. Then, the co-seasonality of rotavirus incidence (which has been shown to have two annual peaks at most MAL-ED sites^28^) and meteorological conditions was adjusted for by including annual and biannual Fourier series sine and cosine functions with site-specific interactions in the model to account for up to two annual peaks.^28^ Rotavirus seasonality patterns differ by location so terms for the interaction between the sites and the annual and biannual harmonics were included, with main effect terms omitted.

Exposures that showed non-linear associations with rotavirus were modelled with restricted cubic spline terms with degrees of freedom ascertained by comparing the Akaike information criterion statistic from models using three to five knots positioned at the corresponding percentiles of the variable distribution. The following potentially confounding covariates were included in all models: vaccine category, age, and sample type.

Vaccine category was a binary variable comparing sites in countries that had introduced the rotavirus vaccine at the time of data collection (Fortaleza, Brazil; Loreto, Peru; and Venda, South Africa) to those that had not (Dhaka, Bangladesh; Vellore, India; Bhaktapur, Nepal; Naushero Feroze, Pakistan; and Haydom, Tanzania) to adjust for the vaccine-related reductions in background transmission levels. Participants' ages (in continuous months) at the time of sample collection were represented in the model by use of linear, quadratic, and cubic terms with vaccine category-specific interaction terms to account for observed differences in the relationship between age and rotavirus transmission in the two groups of sites. Sample type was a binary variable representing whether the sample was a diarrhoeal or monthly surveillance stool included to separately model symptomatic and asymptomatic rotavirus episodes.

Lag lengths for each variable and effect were selected according to criteria outlined in the appendix (p 22) and included with covariates in separate, single-variable models to establish their independent associations with rotavirus. Interactions between these main exposures and stool sample type were included if they were significant at the alpha level of 0·05 since some interactions might differentially affect the probability of inducing symptomatic compared with asymptomatic infections. Probabilities predicted by these single-variable models were plotted across the range of values for each variable, effect, and symptom status. Backward stepwise selection was used to identify variables that retained significance at the alpha level of 0·05 in the presence of the others for each effect. Specific humidity was excluded from all multivariable models because it is almost entirely a function of temperature and relative humidity, and only the results of its independent association are reported. Having selected a model of main effects, forward stepwise selection was used on all possible interactions between retained hydrometeorological variables with each other, by use of multiplicative pairwise combinations of terms, and with vaccine category and sample type with an alpha threshold of 0·01 or lower for inclusion in a final interaction model. Variables that lost significance in the presence of retained interaction terms for other variables were excluded from the final models.

The combined significance of the terms for each covariate—the spline, polynomial, and Fourier series terms and their interactions—were each assessed with the Wald test. To quantify the proportion of the variance explained by the final models and by the meteorological variables and their interactions, full and partial Tjur's Coefficients of Determination (CODs) were calculated from the final models compared with null models that included only the non-hydrometeorological covariates.^29^ The validity of the ability of the two final interaction models to make predictions based on out-of-sample data was assessed by excluding each site in turn, fitting the models to data from the remaining seven sites, then calculating CODs for the excluded site. Analyses were carried out with Stata, version 15.

### Role of the funding source

The funders had no role in study design, study implementation, data analysis, or interpretation of the results. The first author had access to all the data in the study and the corresponding author was responsible for the decision to submit for publication.

## Results

Table 1 summarises the number, proportion, and incidence of rotavirus episodes detected and the number of study participants by sample type, MAL-ED site, and vaccine category. For all sites, both vaccine categories and overall, the percentage of rotavirus-positive samples was higher in diarrhoeal than in monthly stools. For both sample types, the proportion of rotavirus-positive stools was lower for sites in countries where the vaccine had been introduced. However, rotavirus incidence in the Peru site was higher than at several sites that had not yet introduced the vaccine. Additionally, the incidence of diarrhoeal rotavirus in the site in Tanzania, which had not introduced the vaccine, was notably low. A time series needle-plot showing the daily distribution of rotavirus-positive stool samples and durations of follow-up at each site is provided in the appendix (p 1).

**Table 1:** Number of study participants and number, proportion, and incidence of rotavirus-positive samples in each of the MAL-ED study sites, by vaccine category and sample type

	**Monthly samples**			**Diarrhoeal samples**			**Follow-up time (person-years)**	**Total participants**
	Rotavirus-positive samples	Total samples	Rotavirus incidence per 100 person-years	Rotavirus-positive samples	Total samples	Rotavirus incidence per 100 person-years		
**Vaccine introduced**								
Fortaleza, Brazil	38 (1·0%)	3624	10·2	4 (4·4%)	91	1·1	371·7	227
Loreto, Peru	178 (2·8%)	6328	36·9	103 (5·4%)	1892	21·3	482·7	303
Venda, South Africa	104 (1·8%)	5764	20·6	5 (4·6%)	108	1·0	505·3	290
Total	320 (2·0%)	15 716	23·5	112 (5·4%)	2091	8·2	1359·7	820
**Vaccine not yet introduced**								
Dhaka, Bangladesh	264 (5·0%)	5282	57·7	339 (22·3%)	1519	74·1	457·6	265
Vellore, India	374 (6·7%)	5570	80·8	65 (13·4%)	486	14·1	462·6	243
Bhaktapur, Nepal	163 (3·0%)	5519	35·2	95 (11·6%)	822	20·5	462·6	238
Naushero Feroze, Pakistan	172 (3·0%)	5676	33·3	117 (5·5%)	2112	22·7	516·3	275
Haydom, Tanzania	239 (4·6%)	5147	51·2	17 (16·8%)	101	3·6	467·0	259
Total	1212 (4·5%)	27 194	51·2	633 (12·6%)	5040	26·8	2366·0	1280

Data are n (%), unless otherwise indicated.

**Figure 1:**
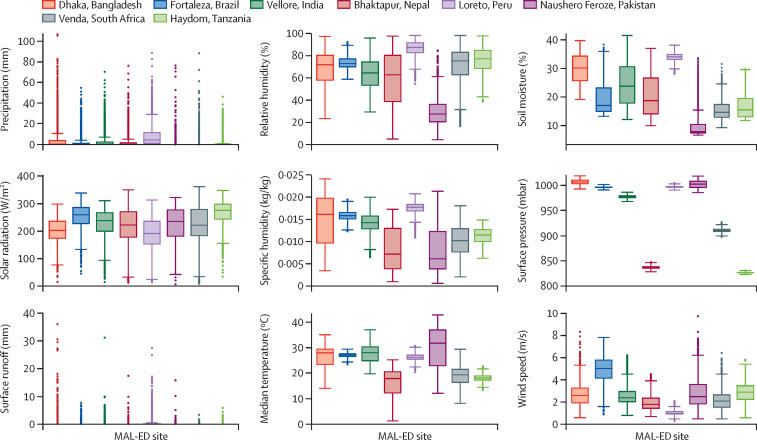
Box plots of the distributions of the nine hydrometeorological variables at the eight MAL-ED sites Global Land Data Assimilation System data are disseminated as part of the mission of NASA's Earth Science Division and archived and distributed by the Goddard Earth Sciences Data and Information Services Center.

Figure 1 shows box plots of the distributions of each GLDAS variable by site, and time series plots of the estimates for these variables at each site from 2009 to 2014 are provided in the appendix (pp 2–10). Precipitation and surface runoff had highly skewed distributions and many days on which the value was zero. Surface pressure values were normally but narrowly distributed within each site, with several showing little or no overlap in their distribution with other sites. High levels of precipitation, surface runoff, soil moisture, and specific humidity were seen at the Bangladesh and Peru sites, reflecting the wet conditions at their locations (an alluvial, deltaic plain subject to annual monsoon in Dhaka, Bangladesh, and a flood-prone confluence of several Amazon tributaries in Loreto, Peru).^16^, ^23^ Low levels of soil moisture, humidity, precipitation, and runoff were observed at the Pakistan site, reflecting the aridity of that location's climate.^22^

The appendix (p 11) shows polynomial smooth plots of the unadjusted association between participants' age in months and the probability of rotavirus positivity for each site and for all sites combined. For many sites, and overall, the association showed an inverse U-shape with peak probability occurring between 6 and 12 months of age, although two of the three vaccine sites (Fortaleza, Brazil; and Loreto, Peru) along with Vellore, India, did not show this pattern. The appendix (p 12) shows equivalent smooth plots for the day of the year on which the sample was collected. As previously reported,^28^ rotavirus transmission was highly seasonal except in the low-transmission Brazil site. The south Asian sites had sharp, primary peaks in the drier period at the turn of the year and a smaller peak in the mid-year monsoon season, while the remaining southern hemisphere sites had mid-year primary peaks and, with the exception of the Peru site, secondary peaks during the wetter part of the year. Equivalent smooth plots for each hydrometeorological exposure are also shown in the appendix (pp 13–21).

**Figure 2:**
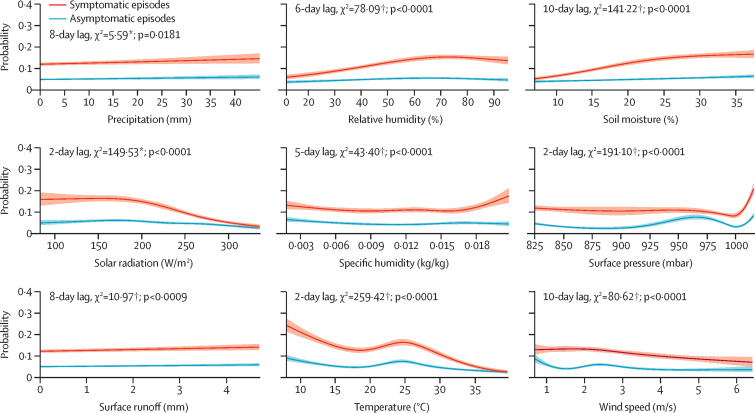
Probabilities of rotavirus infection predicted by single-variable, absolute effect models for nine hydrometeorological variables in the MAL-ED sites Symptomatic (probability of rotavirus positivity for diarrhoeal stool) and asymptomatic (probability of rotavirus positivity for a non-diarrhoeal stool) episodes are shown. *p=0·01 to 0·05. †p<0·0001. For variables for which multiple lags met the criteria for inclusion in subsequent models, only the one with the highest level of significance is shown, but the magnitude and shape of the association did not change substantially for the other lag lengths (not shown).

The results of the lag analysis are described in the appendix (p 23) and described here. Figure 2 plots the probabilities of rotavirus infection predicted by the single-variable absolute effect models by symptom status over the selected lag lengths. The absolute effect of precipitation on rotavirus probability was linear and low in magnitude and significance. Relative humidity and soil moisture both had effects that were significant and similar in magnitude and shape; the direct effect was negligible for asymptomatic infections, but for symptomatic episodes it showed low probability at the bottom extreme of the distribution rising to a plateau in the top tercile. Solar radiation showed a significant inverse association with rotavirus above a value of approximately 190 W/m^2^, most markedly for diarrhoeal episodes. Increasing specific humidity showed no discernible trend in asymptomatic rotavirus infection but for symptomatic episodes a threshold effect with a marked direct association in the upper quartile was observed. The association of surface pressure with symptomatic rotavirus infection also showed a threshold effect with a marked uptick seen at the upper extreme above an approximate value of 1000 mbar, whereas with asymptomatic episodes a secondary peak was observed at approximately 970 mbar. The largest magnitude of effect was for temperature, showing a significant inverse association, with the predicted probability of rotavirus declining from a peak at the cold extreme and a secondary peak at approximately 25°C. Wind speed predicted a steadily declining probability of symptomatic rotavirus infection from a peak at around 2 m/s, but for asymptomatic episodes a sharply decreasing probability from a peak at lowest wind speed was observed. Overall, for the absolute effect model, the risk of rotavirus transmission tended to be higher in wetter and cooler conditions, and with lower solar radiation and wind, suggesting that still atmospheric conditions favour transmission. The pressure results are dominated by site differences, with higher transmission rates found in lower-lying sites. The appendix (p 24) shows the equivalent predicted probabilities when the GLDAS estimates were substituted by ground-based weather station records for those sites and variables for which such data were available (data that have been described in detail elsewhere^12^). The results are broadly consistent with those obtained from the GLDAS exposure data, with slight differences that can be attributed to the incompleteness of the weather station records and other comparative limitations of the two data sources.

**Figure 3:**
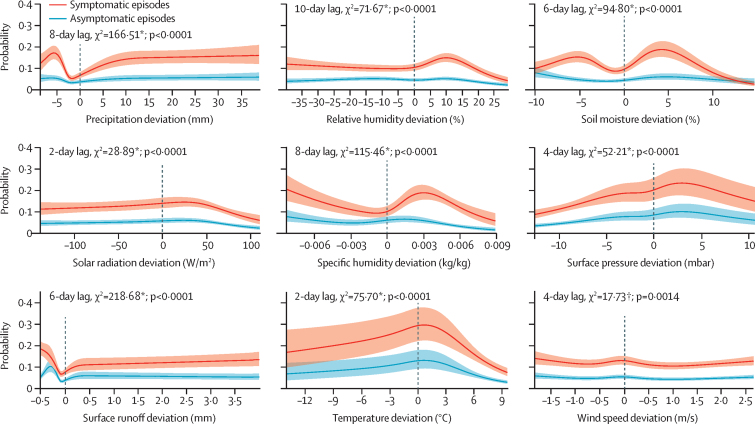
Probabilities of rotavirus infection predicted by single-variable, adjusted effect models for nine hydrometeorological variables in the MAL-ED sites Symptomatic (probability of rotavirus positivity for diarrhoeal stool) and asymptomatic (probability of rotavirus positivity for a non-diarrhoeal stool) episodes are shown. *p<0·0001. †p=0·001 to 0·01. For variables for which multiple lags met the criteria for inclusion in subsequent models, only the one with the highest level of statistical significance is shown, but the magnitude and shape of the association did not change substantial for the other lag lengths (not shown).

Figure 3 shows equivalent plots for the adjusted effects. For the related variables of precipitation and surface runoff, the adjusted effect model predicted significant U-shaped associations in the low extreme, with lowest probability of rotavirus infection just below the site-specific mean value. The adjusted effect of relative humidity on symptomatic rotavirus shows an indirect association below and an inverse U-shaped association above the site-specific mean, with a peak probability at approximately the 75th percentile of the distribution (around 10 percentage points above the site-specific mean). The adjusted effect of soil moisture on symptomatic rotavirus episodes showed a complex, M-shaped association with peaks at approximately 5 percentage points above and below the site-specific mean. Solar radiation, surface pressure, and temperature took on wide, inverse U-shaped associations with peak probabilities occurring slightly above their site-specific mean values. The relationships of temperature and pressure observed in the absolute effects model were not evident in the adjusted model. Instead, risk was highest for average conditions and dropped when weather variability drove either variable away from its average value. The adjusted effect of specific humidity was similar in shape to relative humidity but greater in magnitude and significance. Wind speed was the only variable for which the adjusted association was only moderately significant, a low magnitude effect with a shape that was inverse to that of surface pressure. In contrast to the absolute effect, the adjusted models suggest that somewhat wet conditions—high rainfall generating surface runoff—are associated with a higher risk of rotavirus transmission, whereas relatively low rainfall also brings higher risk. By contrast, day-to-day variability in wetness shown by atmospheric humidity and soil moisture suggests that infection risk is higher under drier conditions across the full range of variability.

**Figure 4:**
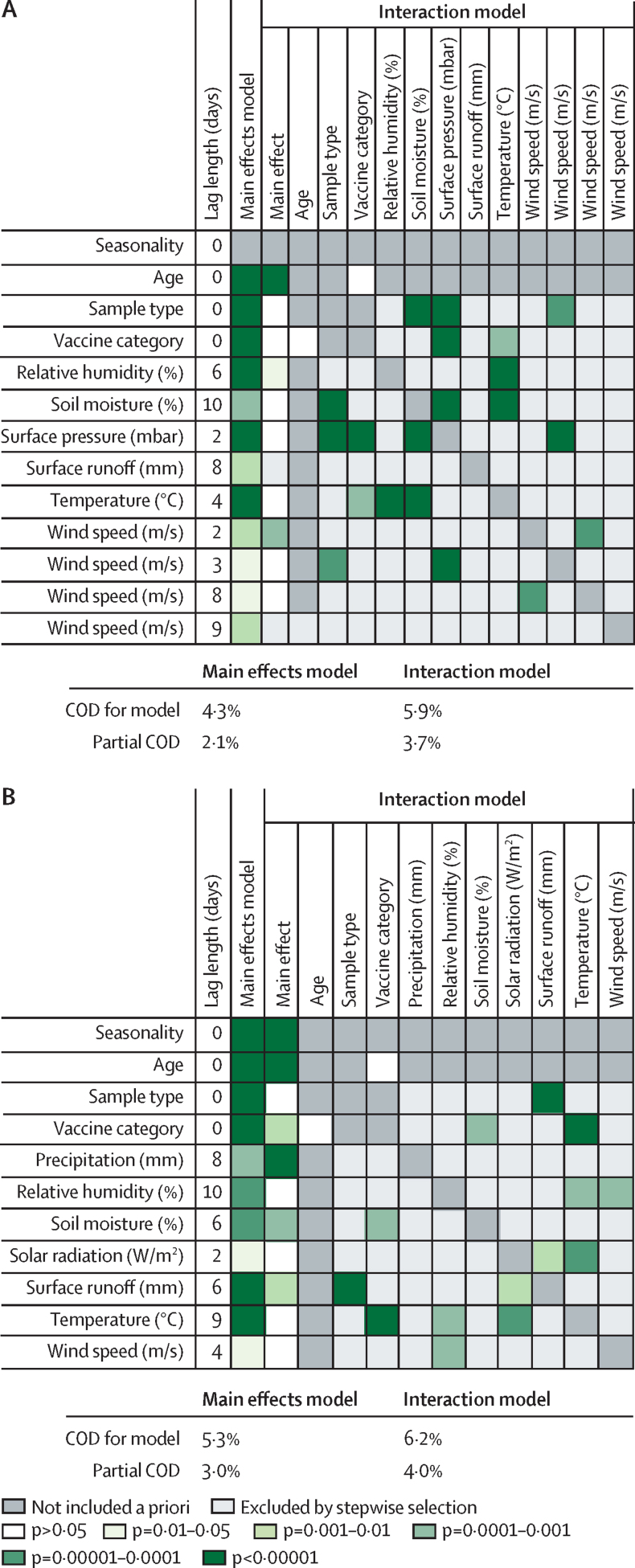
Significance levels from Wald test χ^2^ statistics for associations between hydrometeorological variables, covariates, and their interactions from the final main effect and interaction models (A) absolute effect (absolute values, no adjustment for seasonality). (B) Adjusted effect (site-specific deviations, adjusting for seasonality). COD=Tjur's Coefficients of Determination. Partial COD=COD for included hydrometeorological variables and their interactions.

Figure 4 shows the significance levels for the main effect of each included hydrometeorological variable and for included interactions from both effect models. The risk ratio estimates for all model terms are given in the appendix (pp 25–31). Stepwise selection excluded precipitation, solar radiation, and surface runoff from the final absolute effect interaction model, but not from the final adjusted effect models, from which surface pressure was instead excluded. The final absolute effect model included significant terms for the interaction of sample type with soil moisture, surface pressure, and wind speed, vaccine category with surface pressure and temperature, temperature with relative humidity and soil moisture, surface pressure with soil moisture, and wind speed with surface pressure. Notably, terms for the interaction of wind speed at both 2-day and 8-day lags were also retained in the final absolute effect interaction model. In the final adjusted effect interaction model, interactions between sample type and surface runoff, vaccine category and soil moisture and temperature, and between relative humidity and temperature and wind speed, and between solar radiation and surface runoff and temperature were retained.

The proportion of the variability explained by the final adjusted effect interaction model was higher than for either main effect model or the absolute effect interaction model according to the COD. In absolute terms, however, this proportion was small, with the hydrometeorological variables and their interactions explaining just 4·0% of the variability in rotavirus positivity. Table 2 shows the results of the model validation analysis from withholding observations from each site in turn, treating them as out-of-sample data and calculating the proportion of the variability in rotavirus infection explained by the model at that site. The absolute effect model had low explanatory power for the three sites in countries with the rotavirus vaccine—Brazil, Peru, and South Africa—and the highest explanatory power for the south Asian sites. Most notably, the COD for the Nepal site was markedly higher than at any other site and almost 2·5 times that of the COD for the model as a whole. The ranking of the CODs for the absolute effect among the eight sites roughly corresponded to that of the amplitude of rotavirus seasonality observed at those locations.^28^ The equivalent results for the adjusted model differed considerably from those of the absolute model, although negligible explanatory power was still observed at two vaccine sites (Brazil and South Africa). The CODs for the Bangladesh, India, Peru, and Tanzania sites increased relative to the absolute model, whereas those of the sites in Pakistan and, most markedly, Nepal decreased. In absolute terms the site-specific model predictions had low out-of-sample validity, particularly those of the adjusted effect model, which explained a maximum of just 3·2% of the variability in rotavirus infection status at any one site.

**Table 2:** Site-specific Tjur's Coefficients of Determination for model predictions when observations from each site were withheld and treated as out-of-sample data

	**Absolute effect**	**Adjusted effect**
Dhaka, Bangladesh	0·6%	3·2%
Fortaleza, Brazil	0·3%	0·1%
Vellore, India	0·2%	0·8%
Bhaktapur, Nepal	14·1%	1·1%
Loretu, Peru	0·1%	1·4%
Naushero Feroze, Pakistan	13·1%	1·6%
Venda, South Africa	0·0%	0·2%
Haydom, Tanzania	0·3%	0·9%

Data shown are the proportion of the variability in the rotavirus outcome at each site explained by the final models.

## Discussion

Although previous studies of climate drivers of enteric infectious diseases have incorporated multiple climate variables,^5^ single-day exposure estimates,^7^, ^12^ differential lag relationships,^7^ interactions,^5^ non-linearity,^7^ multiple climate zones,^13^ and adjustment for seasonality,^8^ this analysis is, to the best of our knowledge, the first to address all these factors together. Differences in effect between symptomatic and asymptomatic infections were also assessed. Many hydrometeorological variables—including several not commonly measured by weather stations—showed complex, non-linear associations with rotavirus infection that differed by episode type and were independently and significantly associated across multiple consecutive or non-consecutive lags, including as short a period as 2 days. The results show that the effect of climate on rotavirus transmission is being mediated by four independent mechanisms, shown in the appendix (p 32): waterborne dispersal, airborne dispersal, survival on soil and surfaces, and host factors.

With regard to waterborne dispersal, it is thought that precipitation drives enteric pathogen transmission via different mechanisms at the two extremes, with heavy rainfall and runoff flushing microorganisms from soils and surfaces into water sources and drought conditions, concentrating them in these environments.^4^ The U-shaped association between precipitation and rotavirus infection predicted by the adjusted effect model, with lowest probability at the site-specific average, supports this hypothesis, since small-scale water and drainage systems that handle precipitation and runoff levels within the typical range for a particular location might be overwhelmed by relative extremes that would be normal in other places. Furthermore, that the absolute effect of precipitation was barely significant and that this variable was excluded from the final absolute effect model by stepwise selection might be due to these two competing pathways (the runoff effect versus the concentration effect^4^) influencing rotavirus transmission in opposite directions in the rainy season compared with drier times of the year, which could cancel each other out unless seasonality is adjusted for. Recent studies have found that these opposing effects, each taking over from the other as rainy seasons give way to drier periods, are a notable feature of rotavirus epidemiology, particularly in southeast Asia.^28^, ^30^ The absence of an interaction identified between precipitation and any other hydrometeorological variable is consistent with this variable operating via a separate pathway. That the final adjusted effect model included solar radiation might be because it acts as a surrogate for precipitation, since values for this variable will be lower on rainy, overcast days when cloud cover obscures sunlight.

An airborne route of transmission for rotavirus has long been suspected but not conclusively proven.^31^ Hypothesised vehicles for this route of transmission include either dried dust^14^ or liquid particles^5^ that might either be ingested directly^32^ or inhaled first, before migrating to the gastrointestinal tract through the swallowing of respiratory secretions.^33^ Aerosol transmission of this kind is to be distinguished from droplet transmission by the smaller size of the contaminated particles involved (<5 μm diameter according to WHO's definition), which permits these particles to remain suspended in the air for days at a time, settling at a rate that is a function both of their size and the movement of the air and being capable of infecting susceptible individuals at a greater distance from their source.^34^ Although aerosol transmission is usually associated with particles secreted from the respiratory tract, formation of aerosols can also occur from disposal of excreta in nappies and through toilet flushing.^5^ Once suspended, environmental conditions affect the ability of pathogens to survive and remain infectious in aerosols by determining the size of the particles and the rate at which they desiccate.^34^

Factors on this pathway that were assessed in this analysis include temperature, humidity, pressure, and wind speed, a cluster of closely-interrelated variables that were all retained in at least one final effect model and showed considerable mutual interaction. The strong association between surface pressure and rotavirus transmission in the absolute effect model might be due to a direct effect on aerosol dynamics, or because GLDAS estimates of pressure are more strongly correlated with true, ground-level advective air movement than are estimates of wind speed at a 10 m height. In the adjusted model, the predicted probability of rotavirus was highest at site-specific mean pressure, at which stiller conditions would be expected. This observation is consistent with the negative relationship with wind speed seen in the absolute effect models, suggesting that transmission is promoted when aerosols are able to linger in slow moving air and inhibited when stronger winds transport particles away from susceptible individuals. This finding is in contrast to a previously reported direct relationship with wind speed^5^ and therefore merits further investigation, especially given the apparent importance of wind speed and its interaction with other variables in both final models.

The direct association between absolute values of relative humidity and rotavirus appeared to reverse into a broadly negative relationship when these were substituted for site-specific deviations. This observation might be due to rotavirus transmission being higher in MAL-ED sites where conditions are broadly more humid. The within-site effect of relative humidity is consistent with dry dust particles being a vehicle for aerosol transmission, since a peak in the probability of infection was seen at lowest humidity. However, the second peak (slightly larger in magnitude in the seasonality-adjusted model) at around 10 percentage points above the site-specific mean relative humidity might indicate that liquid aerosols also have a role. The low probability of infection at high relative humidity might be because aerosols are unable to suspend for long periods in moisture-dense air. Similar changes in the shape of the within-site effect compared to the absolute effect were seen for specific humidity and soil moisture. Temperature is also thought to affect the size and dynamics of infectious airborne particles.^35^ In the adjusted effect model, we identified an apparent peak in rotavirus transmission at mean temperature, with decreasing risk towards the upper extreme of the location-specific temperature distribution.

Soil moisture was consistently significant across effects and lag lengths, remaining so in the presence of precipitation and relative humidity in the final models, suggesting that it was not merely acting as an indicator of generally moist conditions but had its own independent effect. Non-linear effects of the viability of enteric viruses in soil at varying moisture levels have been documented,^36^ as has increased persistence of rotavirus on surfaces and fomites at both higher^37^ and lower^38^ humidity. Once airborne rotavirus-contaminated aerosols settle, the virus might remain viable for longer if it then adheres to surfaces that permit retention of its surrounding moisture^5^, interacting with the airborne dispersal route as indicated in the absolute and adjusted effect interaction models.

Although the absolute effect indicated a linear, direct association, with higher soil moisture causing a higher risk of rotavirus transmission, the within-site effect suggests a more complex, M-shaped relationship, where soil moisture values of around 5 percentage points above and 5 percentage points below the site-specific median are associated with the highest risk of rotavirus transmission. A possible interpretation of this observed association is that rotavirus survival is optimised on soil that is dry enough to form dust particles but not so dry as to desiccate entirely, or moist enough for adhered viruses to remain viable, but not so moist that they are flushed from the soil.

With regard to host factors, the absolute effect model for temperature predicted high rotavirus transmission occurring at the coldest extreme of the distribution; however, in the seasonality-adjusted model, the effect took on an inverse U-shape. A possible explanation for this observation is that, at colder times of the year, increased contact rates between susceptible and infected individuals as they congregate indoors promotes rotavirus transmission, a confounding effect that can be removed by adjusting for seasonality. However, in most sites included in this study, the temperature seldom fell below 15°C, so if such an effect existed it is unlikely that it would be particularly strong. A role for UV radiation has been suggested in the downregulation of the immune system, a host-mediated factor that could exacerbate the course and severity of viral infections.^5^ This hypothesis is supported by the fact that solar radiation remains significantly associated with rotavirus infection after adjustment for seasonality; however, we consider it more likely that this superficial association represents confounding due to rainclouds obscuring the sun on rainy days, rather than any direct, biological effect.

The low CODs for the final models and for the site-specific out-of-sample model predictions reflect the large proportion of the variability left unexplained by the final models (although there is some indication that predictions from the absolute effect model might have relatively higher explanatory power in sites with a higher amplitude of rotavirus seasonality). This is to be expected but might undermine confidence in the replicability of this study's findings and should be considered by researchers attempting to model rotavirus transmission for predicting and projecting future disease burden. Hydrometeorological predictors are likely to be of low explanatory power and minimal clinical significance compared with household, behavioural, and immunological factors. Future analyses could adjust for easily measured, non-environmental exposures (such as breastfeeding status and sanitation) to increase predictive power. However, identification of the individual-level factors most likely to ascertain disease risk (such as fucosyltransferase 2 phenotype^39^) might be unfeasible at this scale. Modelling interactions between hydrometeorological variables can produce modest improvements, as demonstrated by the increase in COD for the interaction models relative to the main effects.

This analysis has several limitations. Some of these are inherent to the model-based EO exposure data, which, despite their advantages of spatial and temporal completeness and mutual consistency among variables, have levels of uncertainty that vary widely and have seldom been validated for rural areas, where precision of estimates is most lacking.^12^ It has been recommended that, as a sensitivity analysis, results of epidemiological studies that use EO-derived exposures should be compared with those obtained from ground-based measurements whenever possible to triangulate the true effect.^12^ This study adhered to this practice and found broad consistency between the two data sources. The analysis did not, however, consider the role of individual behavioural or household-level factors, since these were outside the scope of the study. As noted above, these individual behavioural or household-level factors might be stronger determinants of rotavirus transmission risk, but they are more difficult to measure at a population level. To apply a consistent approach to all variables, this analysis did not consider the cumulative effect of variables aggregated over multiday periods (eg, 7-day total precipitation volume of average temperature) since this went beyond the scope of the study objective to ascertain non-arbitrary, biologically relevant time windows for each variable. The results of the lag analysis indicate that such potential cumulative effects might be a fruitful subject of future analyses of the same data for particular variables of interest.

Finally, an inevitable limitation of this study is the level of geographical representativeness that can be provided by including just eight locations, which restricts the generalisability of these results to other climate zones. However, since the EO datasets are available at a global scale and subdaily resolution, and updated continually, similar studies at different locations can be added to derive more precise predictions for more diverse conditions. Furthermore, emerging tools for objective climate regionalisation can be combined with the results to divide extensive geographical zones into smaller regions that are homogeneous with respect to important climate characteristics.^40^

## References

[1] Wu X, Lu Y, Zhou S, Chen L, Xu B (2016). Impact of climate change on human infectious diseases: Empirical evidence and human adaptation. Environ Int.

[2] Liu L, Oza S, Hogan D (2016). Global, regional, and national causes of under-5 mortality in 2000–15: an updated systematic analysis with implications for the Sustainable Development Goals. Lancet.

[3] WHO (2014). Quantitative risk assessment of the effects of climate change on selected causes of death, 2030s and 2050s. http://www.who.int/globalchange/publications/quantitative-risk-assessment/en/.

[4] Levy K, Woster AP, Goldstein RS, Carlton EJ (2016). Untangling the Impacts of climate change on waterborne diseases: a systematic review of relationships between diarrheal diseases and temperature, rainfall, flooding, and drought. Environ Sci Technol.

[5] Hervás D, Hervás-Masip J, Rosell A, Mena A, Pérez JL, Hervás JA (2014). Are hospitalizations for rotavirus gastroenteritis associated with meteorologic factors?. Eur J Clin Microbiol Infect Dis.

[6] Lanata CF, Fischer-Walker CL, Olascoaga AC (2013). Global causes of diarrheal disease mortality in children. PLoS One.

[7] Hashizume M, Armstrong B, Wagatsuma Y, Faruque ASG, Hayashi T, Sack DA (2008). Rotavirus infections and climate variability in Dhaka, Bangladesh: a time-series analysis. Epidemiol Infect.

[8] D'Souza RM, Hall G, Becker NG (2008). Climatic factors associated with hospitalizations for rotavirus diarrhoea in children under 5 years of age. Epidemiol Infect.

[9] Sumi A, Rajendran K, Ramamurthy T (2013). Effect of temperature, relative humidity and rainfall on rotavirus infections in Kolkata, India. Epidemiol Infect.

[10] Ijaz MK, Sattar SA, Johnson-Lussenburg CM, Springthorpe VS, Nair RC (1985). Effect of relative humidity, atmospheric temperature, and suspending medium on the airborne survival of human rotavirus. Can J Microbiol.

[11] Chan T-C, Fu Y-C, Hwang J-S (2015). Changing social contact patterns under tropical weather conditions relevant for the spread of infectious diseases. Epidemiol Infect.

[12] Colston JM, Ahmed T, Mahopo C (2018). Evaluating meteorological data from weather stations, and from satellites and global models for a multi-site epidemiological study. Environ Res.

[13] Jagai JS, Sarkar R, Castronovo D (2012). Seasonality of rotavirus in South Asia: a meta-analysis approach assessing associations with temperature, precipitation, and vegetation index. PLoS One.

[14] Levy K, Hubbard AE, Eisenberg JNS (2009). Seasonality of rotavirus disease in the tropics: a systematic review and meta-analysis. Int J Epidemiol.

[15] MAL-ED Network Investigators (2014). The MAL-ED study: a multinational and multidisciplinary approach to understand the relationship between enteric pathogens, malnutrition, gut physiology, physical growth, cognitive development, and immune responses in infants and children up to 2 years of. Clin Infect Dis.

[16] Ahmed T, Mahfuz M, Islam MM (2014). The MAL-ED cohort study in Mirpur, Bangladesh. Clin Infect Dis.

[17] Bessong PO, Nyathi E, Mahopo TC, Netshandama V, MAL-ED South Africa (2014). Development of the Dzimauli community in Vhembe District, Limpopo province of South Africa, for the MAL-ED cohort study. Clin Infect Dis.

[18] John SM, Thomas RJ, Kaki S (2014). Establishment of the MAL-ED birth cohort study site in Vellore, Southern India. Clin Infect Dis.

[19] Lima AAM, Oriá RB, Soares AM (2014). Geography, population, demography, socioeconomic, anthropometry, and environmental status in the MAL-ED cohort and case-control study Sites in Fortaleza, Ceará, Brazil. Clin Infect Dis.

[20] Mduma ER, Gratz J, Patil C (2014). The etiology, risk factors, and interactions of enteric infections and malnutrition and the consequences for child health and development study (MAL-ED): description of the Tanzanian site. Clin Infect Dis.

[21] Shrestha PS, Shrestha SK, Bodhidatta L (2014). Bhaktapur, Nepal: The MAL-ED Birth Cohort Study in Nepal. Clin Infect Dis.

[22] Turab A, Soofi SB, Ahmed I, Bhatti Z, Zaidi AKM, Bhutta ZA (2014). Demographic, socioeconomic, and health characteristics of the MAL-ED network study site in rural Pakistan. Clin Infect Dis.

[23] Yori PP, Lee G, Olortegui MP (2014). Santa Clara de Nanay: the MAL-ED Cohort in Peru. Clin Infect Dis.

[24] Liu J, Gratz J, Amour C (2016). Optimization of quantitative PCR methods for enteropathogen detection. PLoS One.

[25] Houpt E, Gratz J, Kosek M (2014). Microbiologic methods utilized in the MAL-ED cohort study. Clin Infect Dis.

[26] Rodell M, Houser PR, Jambor U (2004). The global land data assimilation system. Bull Amer Meteor Soc.

[27] Zou G (2004). A Modified Poisson regression approach to prospective studies with binary data. Am J Epidemiol.

[28] Colston JM, Ahmed AMS, Soofi SB (2018). Seasonality and within-subject clustering of rotavirus infections in an eight-site birth cohort study. Epidemiol Infect.

[29] Tjur T (2009). Coefficients of determination in logistic regression models—a new proposal: the coefficient of Discrimination. Am Stat.

[30] Hasan MA, Mouw C, Jutla A, Akanda AS (2018). Quantification of rotavirus diarrheal risk due to hydroclimatic extremes over South Asia: prospects of satellite-based observations in detecting outbreaks. GeoHealth.

[31] Dennehy PH (2000). Transmission of rotavirus and other enteric pathogens in the home. Pediatr Infect Dis J.

[32] Barril PA, Fumian TM, Prez VE (2015). Rotavirus seasonality in urban sewage from Argentina: effect of meteorological variables on the viral load and the genetic diversity. Environ Res.

[33] Prince DS, Astry C, Vonderfecht S, Jakab G, Shen F-M, Yolken RH (1986). Aerosol transmission of experimental rotavirus infection. Pediatr Infect Dis J.

[34] Jones RM, Brosseau LM (2015). Aerosol transmission of infectious disease. J Occup Environ Med.

[35] Fernstrom A, Goldblatt M (2013). Aerobiology and its role in the transmission of infectious diseases. J Pathog.

[36] Hurst CJ, Gerba CP, Cech I (1980). Effects of environmental variables and soil characteristics on virus survival in soil. Appl Environ Microbiol.

[37] Abad FX, Pintó RM, Bosch A (1994). Survival of enteric viruses on environmental fomites. Appl Environ Microbiol.

[38] Ansari SA, Springthorpe VS, Sattar SA (1991). Survival and vehicular spread of human rotaviruses: possible relation to seasonality of outbreaks. Rev Infect Dis.

[39] Colston JM, Francois R, Pisanic N (2019). Effects of child and maternal Histo Blood Group Antigen status on symptomatic and asymptomatic enteric infections in early childhood. J Infect Dis.

[40] Badr HS, Zaitchik BF, Dezfuli AK Climate regionalization through hierarchical clustering: options and recommendations for Africa. American Geophysical Union, Fall Meeting 2014. Abstract GC53B-0538 2014. http://adsabs.harvard.edu/abs/2014AGUFMGC53B0538B.

