# Supplementary material for: Use of earth observation-derived hydrometeorological variables to model and predict rotavirus infection (MAL-ED): a multisite cohort study
Source: Lancet Planet Health. 2019 Jun;3(6):e248–58. doi: 10.1016/S2542-5196(19)30084-1 (PMC6650544; doi:10.1016/S2542-5196(19)30084-1)
Supplement: Supplementary appendix [file mmc1.pdf]

THE LANCET
Planetary Health

Supplementary appendix

This appendix formed part of the original submission and has been peer reviewed. We post it as supplied by the authors.

Supplement to: Colston J M, Zaitchik B, Kang K, et al. Use of earth observation-derived hydrometeorological variables to model and predict rotavirus infection (MAL-ED): a multisite cohort study. *Lancet Planet Health* 2019: **3:** e248–58.

Using earth observation-derived hydrometeorological variables to model and predict the probability of rotavirus infection in an eight-site cohort study, J. M. Colston et al. 2019, *The Lancet Planetary Health*

Supplementary materials:

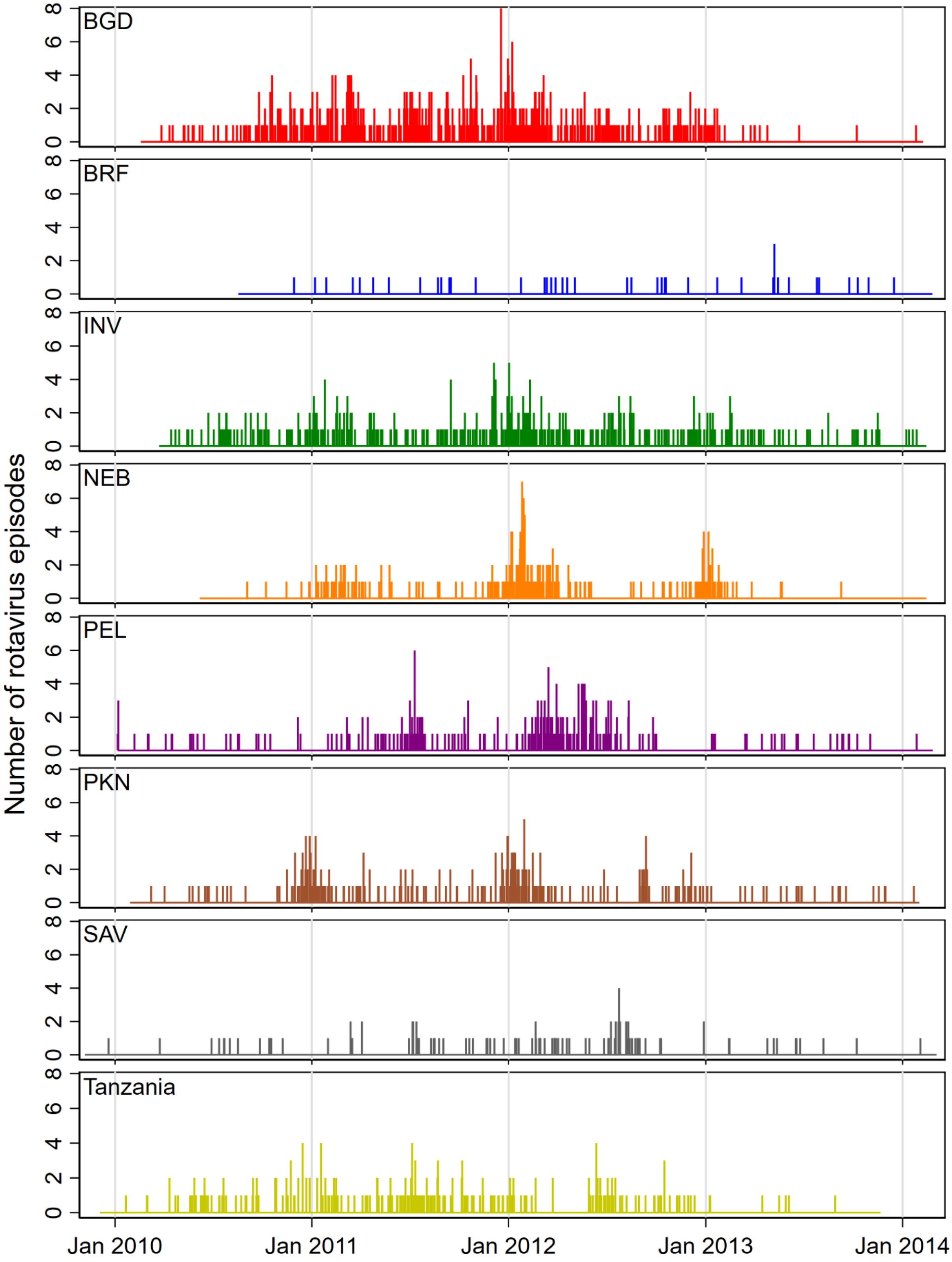

Figure S1: Time series needle plot of the daily distribution of rotavirus-positive stool samples recorded at each MAL-ED site (rotavirus-negative samples not shown)

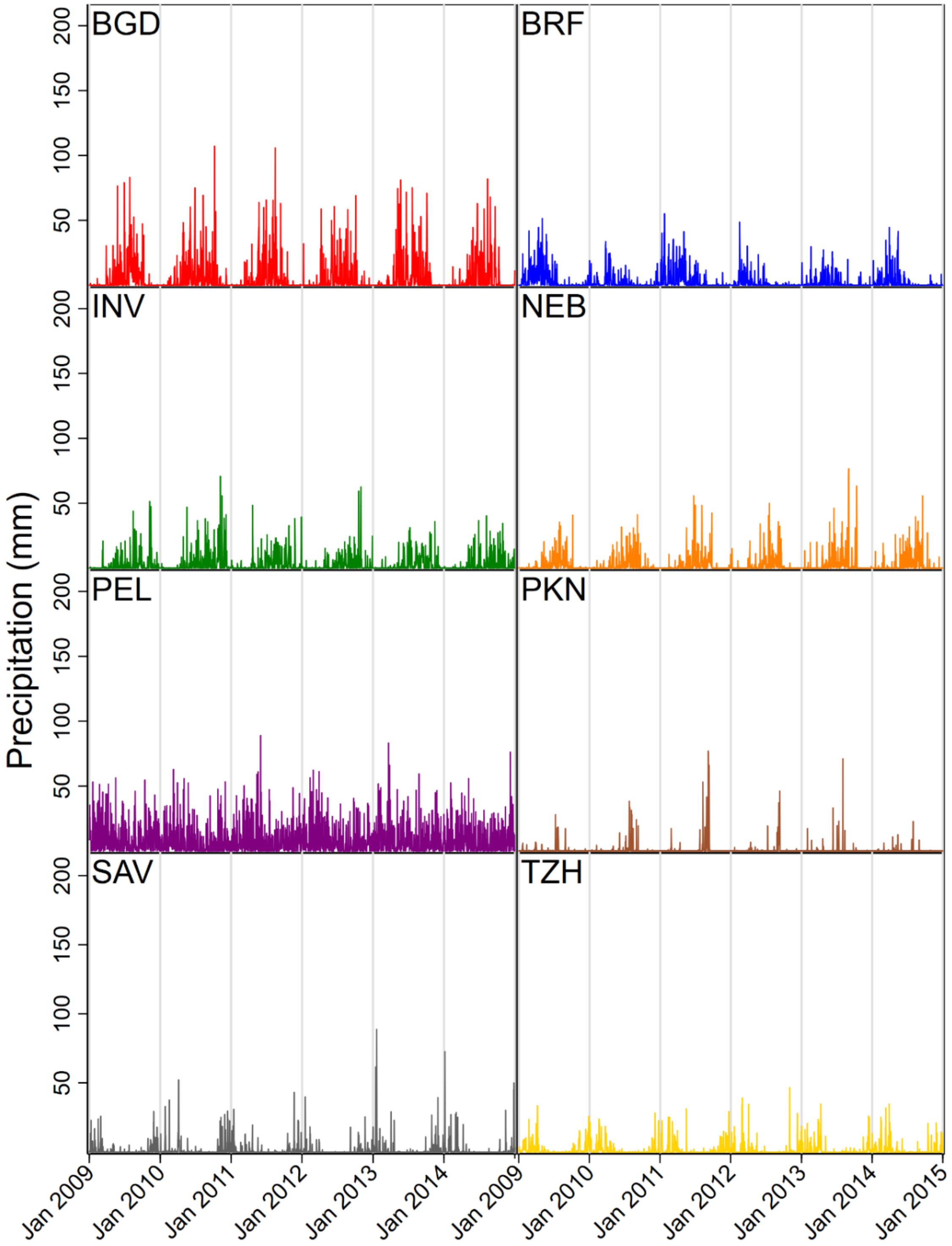

Figure S2: Daily total precipitation estimates from GLDAS at the eight MAL-ED sites, 2009 – 2014[1]

[1] BGD = Dhaka, Bangladesh; BRF = Fortaleza, Brazil; INV = Vellore, India; NEB = Bhaktapur, Nepal; PKN = Naushero Feroze Pakistan; PEL = Loreto, Peru; SAV = Venda, South Africa; TZH = Haydom, Tanzania. Adapted from Colston et al. 2018

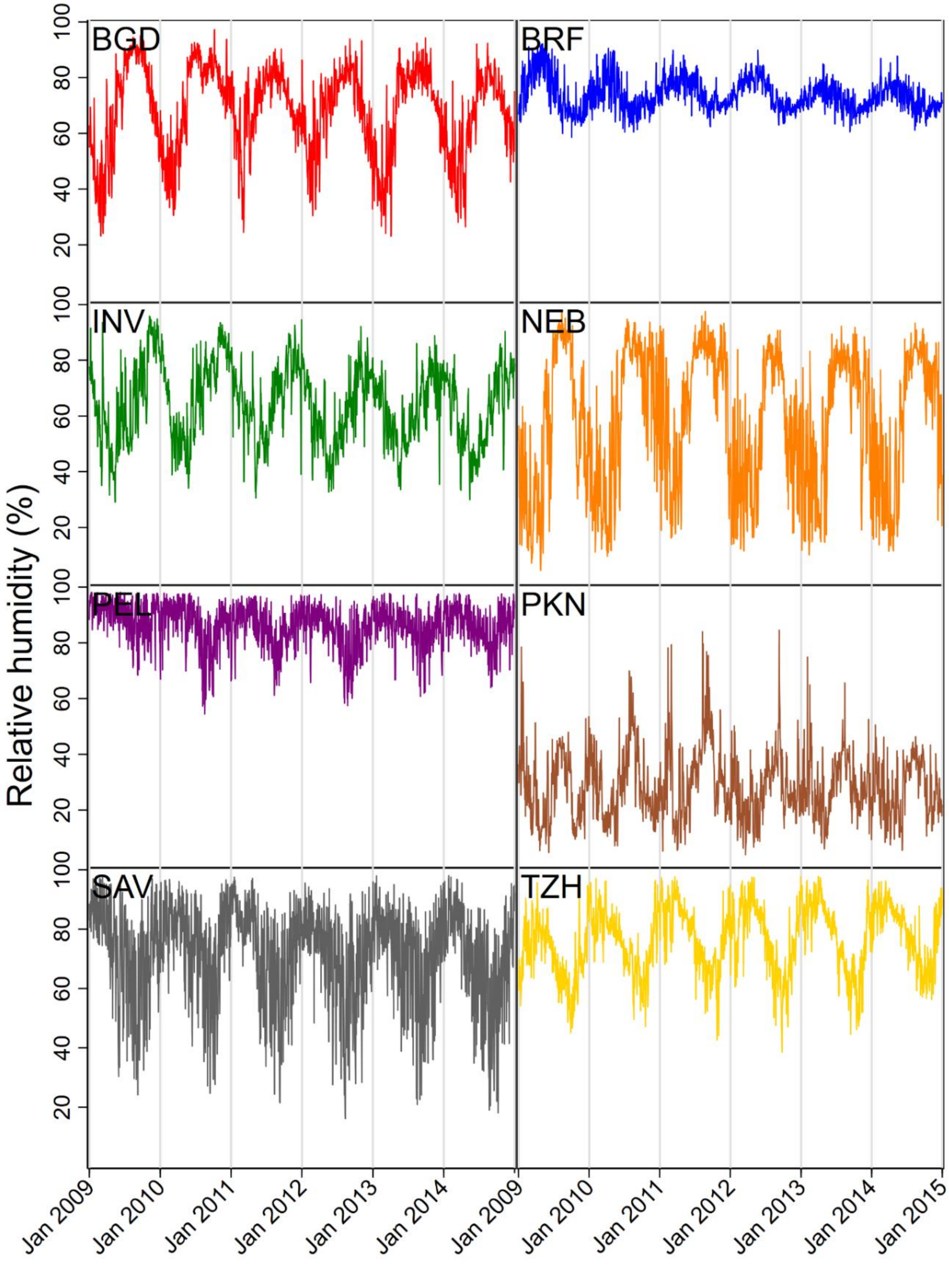

Figure S3: Daily average relative humidity estimates from GLDAS at the eight MAL-ED sites, 2009 - 2014[2]

[2] BGD = Dhaka, Bangladesh; BRF = Fortaleza, Brazil; INV = Vellore, India; NEB = Bhaktapur, Nepal; PKN = Naushero Feroze Pakistan; PEL = Loreto, Peru; SAV = Venda, South Africa; TZH = Haydom, Tanzania. Adapted from Colston et al. 2018

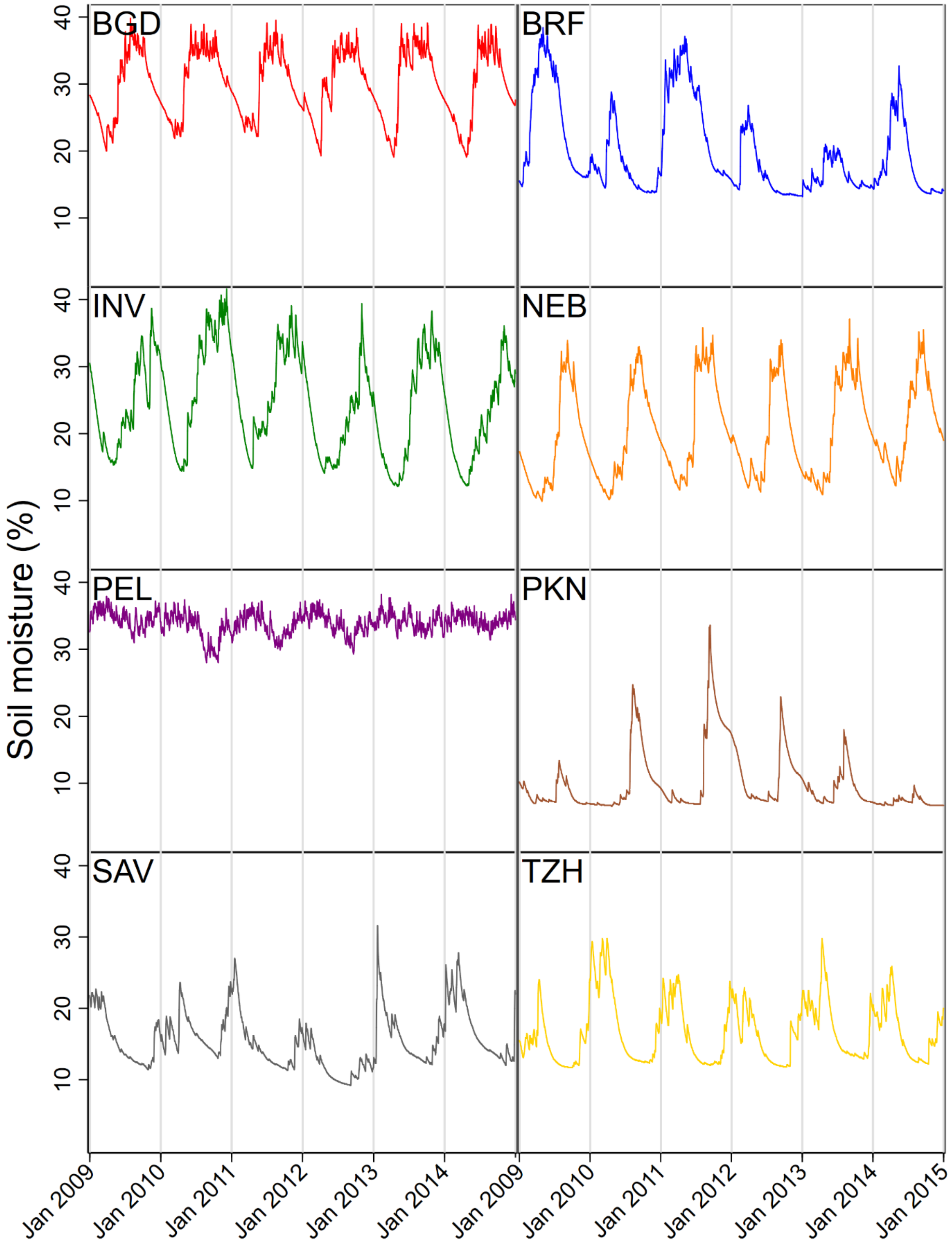

Figure S4: Daily average soil moisture estimates from GLDAS at the eight MAL-ED sites, 2009 – 2014[3]

[3] BGD = Dhaka, Bangladesh; BRF = Fortaleza, Brazil; INV = Vellore, India; NEB = Bhaktapur, Nepal; PKN = Naushero Feroze Pakistan; PEL = Loreto, Peru; SAV = Venda, South Africa; TZH = Haydom, Tanzania. Adapted from Colston et al. 2018

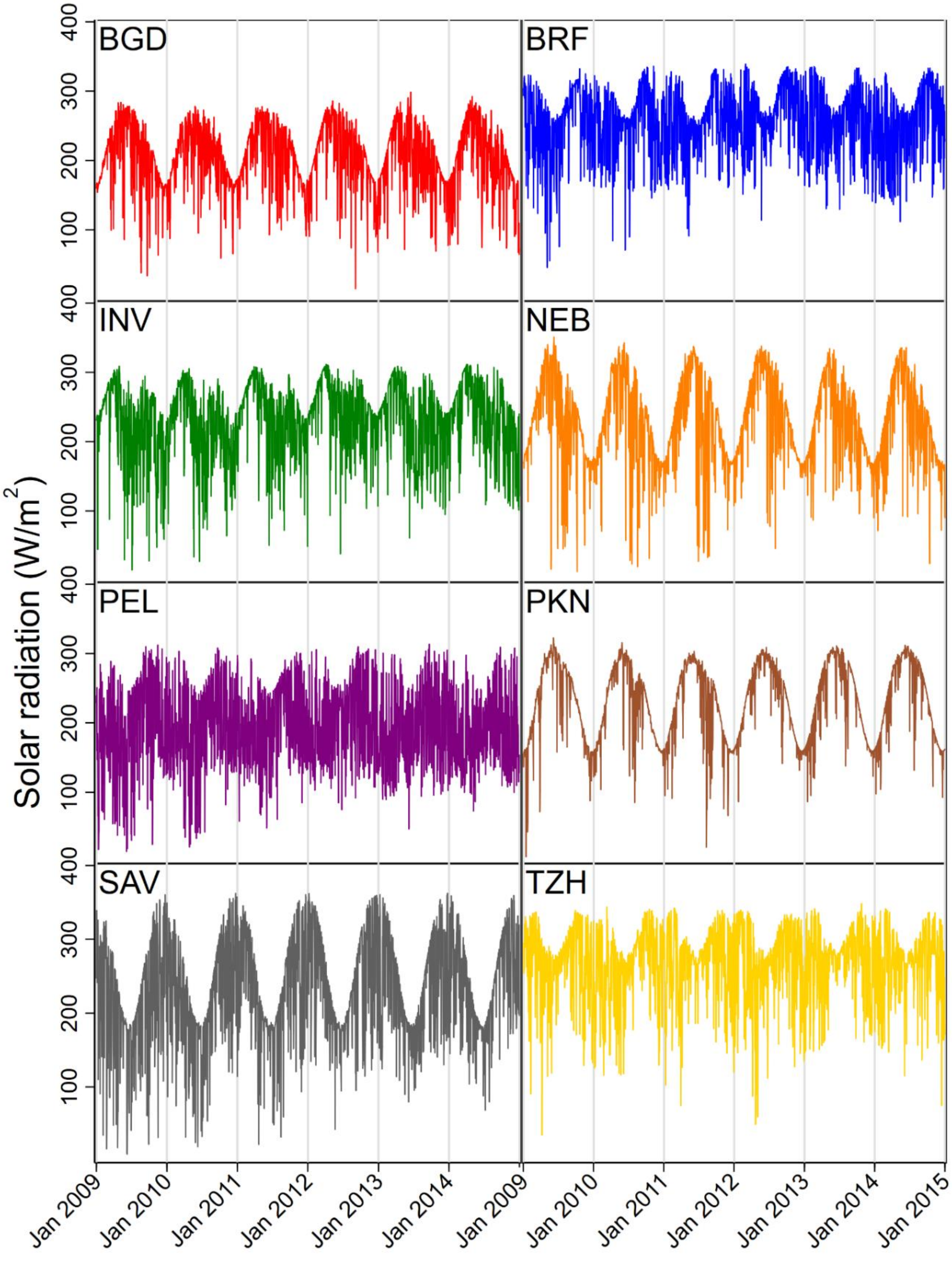

Figure S5: Daily average solar radiation estimates from GLDAS at the eight MAL-ED sites, 2009 – 2014[4]

[4] BGD = Dhaka, Bangladesh; BRF = Fortaleza, Brazil; INV = Vellore, India; NEB = Bhaktapur, Nepal; PKN = Naushero Feroze Pakistan; PEL = Loreto, Peru; SAV = Venda, South Africa; TZH = Haydom, Tanzania. Adapted from Colston et al. 2018

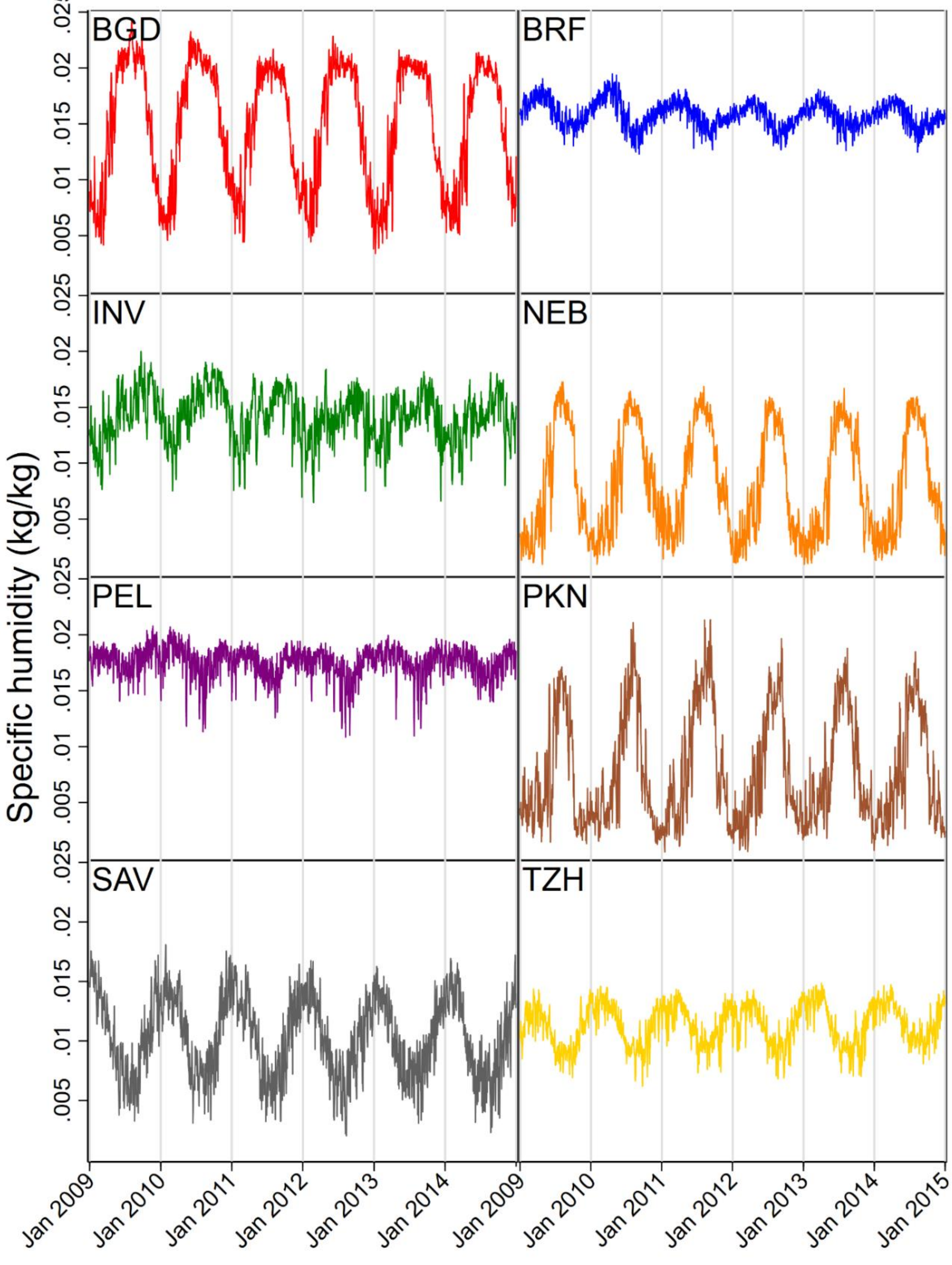

Figure S6: Daily average specific humidity estimates from GLDAS at the eight MAL-ED sites, 2009 – 2014[5]

---

[5] BGD = Dhaka, Bangladesh; BRF = Fortaleza, Brazil; INV = Vellore, India; NEB = Bhaktapur, Nepal; PKN = Naushero Feroze Pakistan; PEL = Loreto, Peru; SAV = Venda, South Africa; TZH = Haydom, Tanzania. Adapted from Colston et al. 2018

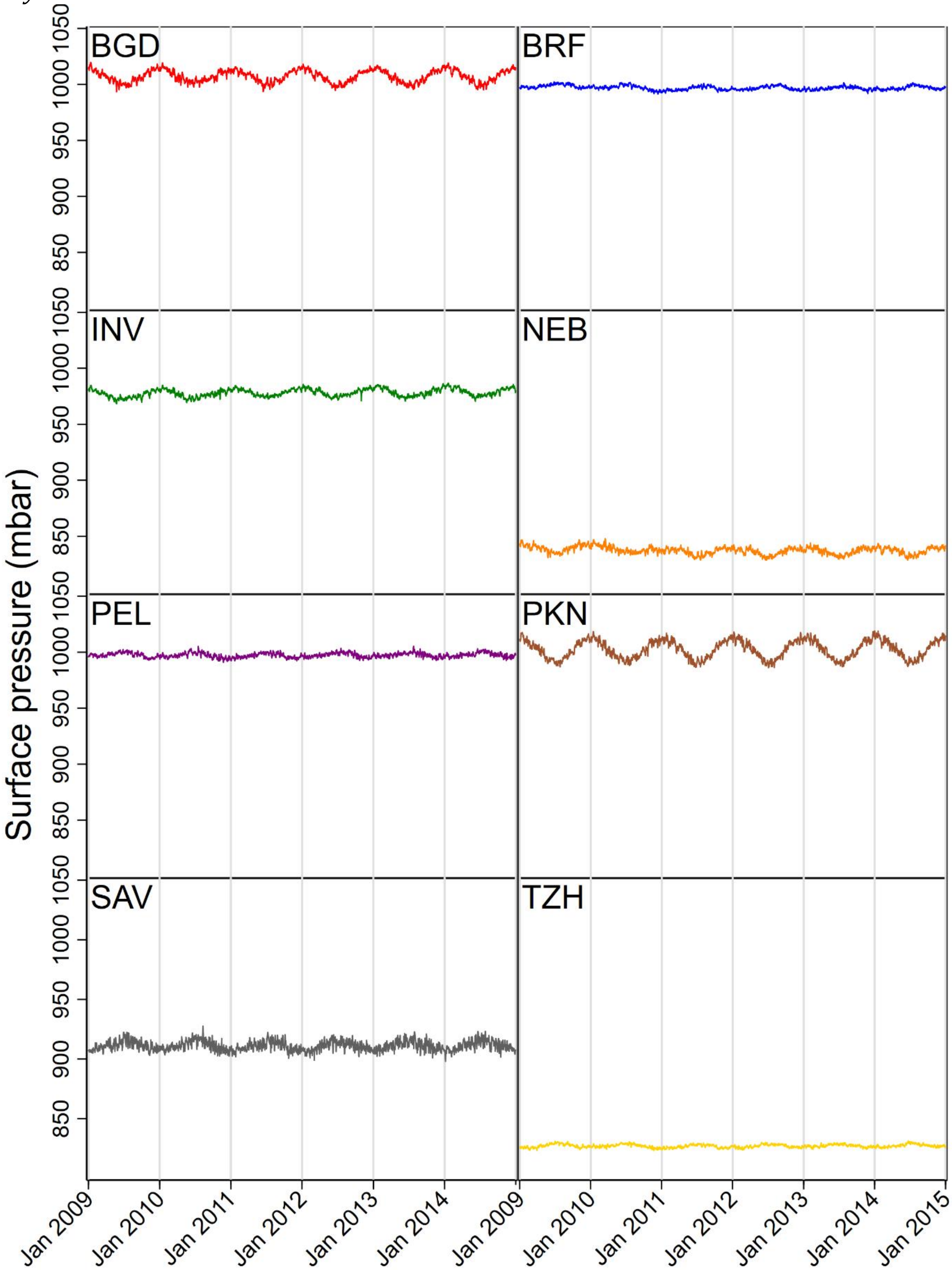

Figure S7: Daily average surface pressure estimates from GLDAS at the eight MAL-ED sites, 2009 – 2014[6]

[6] BGD = Dhaka, Bangladesh; BRF = Fortaleza, Brazil; INV = Vellore, India; NEB = Bhaktapur, Nepal; PKN = Naushero Feroze Pakistan; PEL = Loreto, Peru; SAV = Venda, South Africa; TZH = Haydom, Tanzania. Adapted from Colston et al. 2018

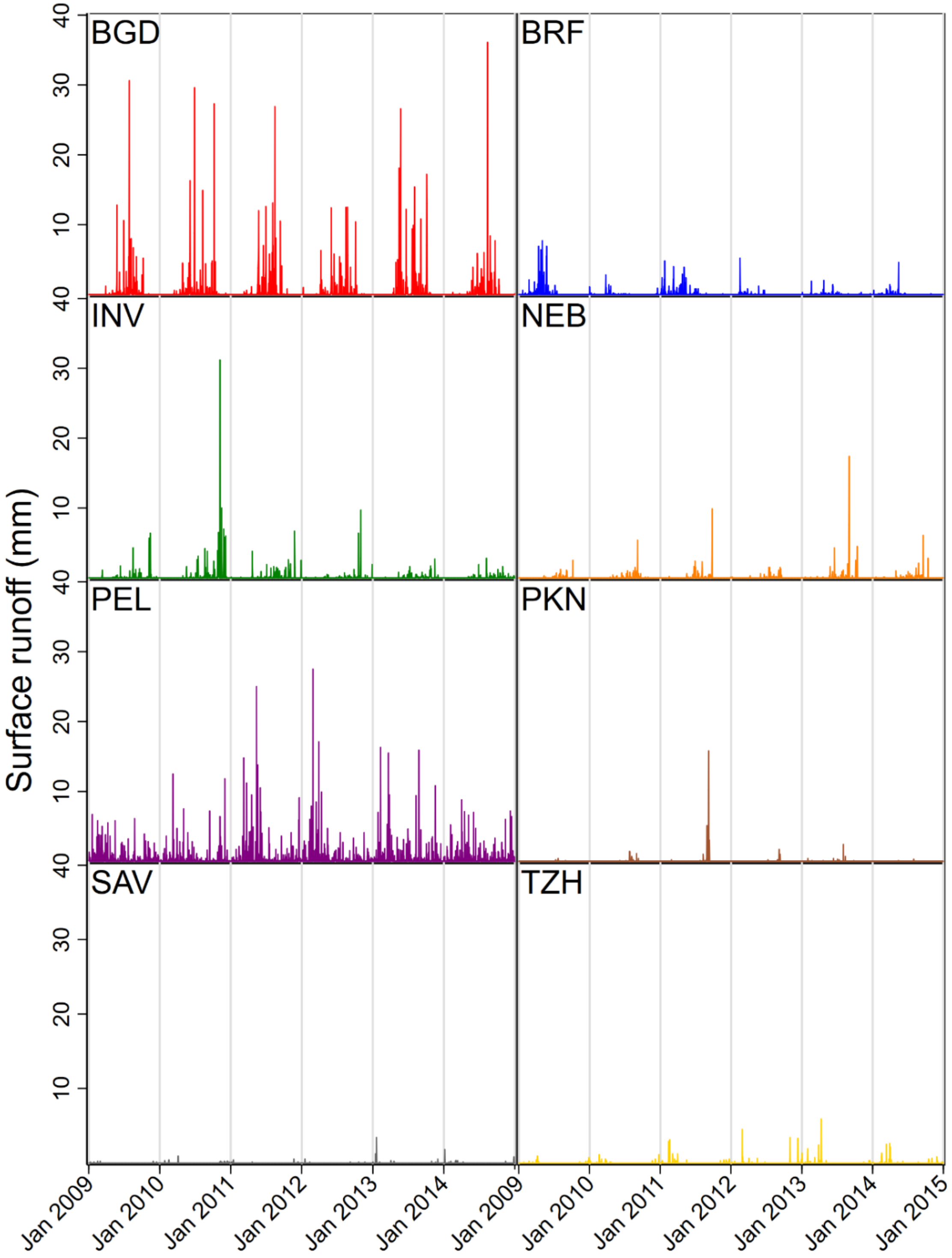

Figure S8: Daily total surface runoff estimates from GLDAS at the eight MAL-ED sites, 2009 – 2014[7]

[7] BGD = Dhaka, Bangladesh; BRF = Fortaleza, Brazil; INV = Vellore, India; NEB = Bhaktapur, Nepal; PKN = Naushero Feroze Pakistan; PEL = Loreto, Peru; SAV = Venda, South Africa; TZH = Haydom, Tanzania. Adapted from Colston et al. 2018

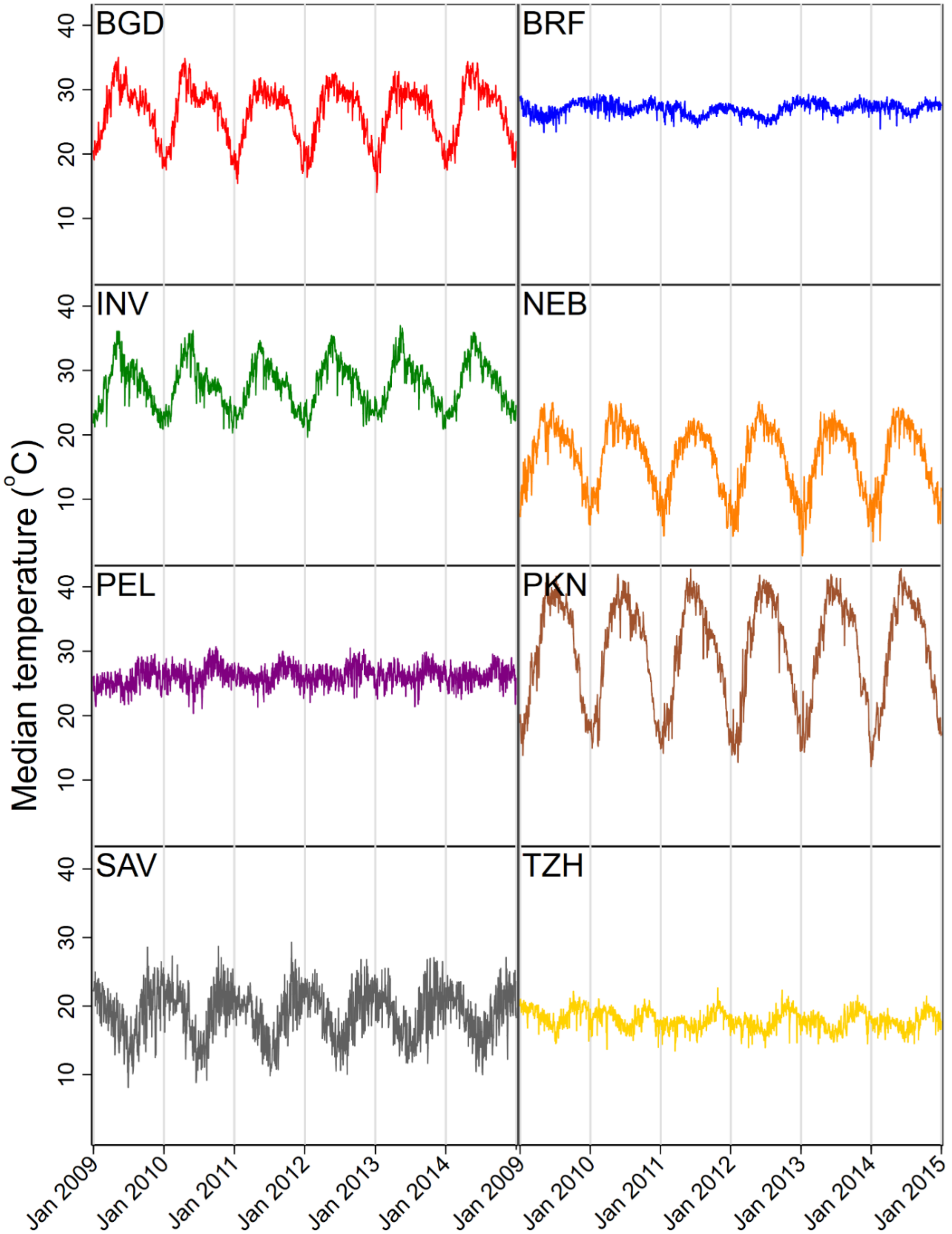

Figure S9: Daily average temperature estimates from GLDAS at the eight MAL-ED sites, 2009 – 2014[8]

[8] BGD = Dhaka, Bangladesh; BRF = Fortaleza, Brazil; INV = Vellore, India; NEB = Bhaktapur, Nepal; PKN = Naushero Feroze Pakistan; PEL = Loreto, Peru; SAV = Venda, South Africa; TZH = Haydom, Tanzania. Adapted from Colston et al. 2018

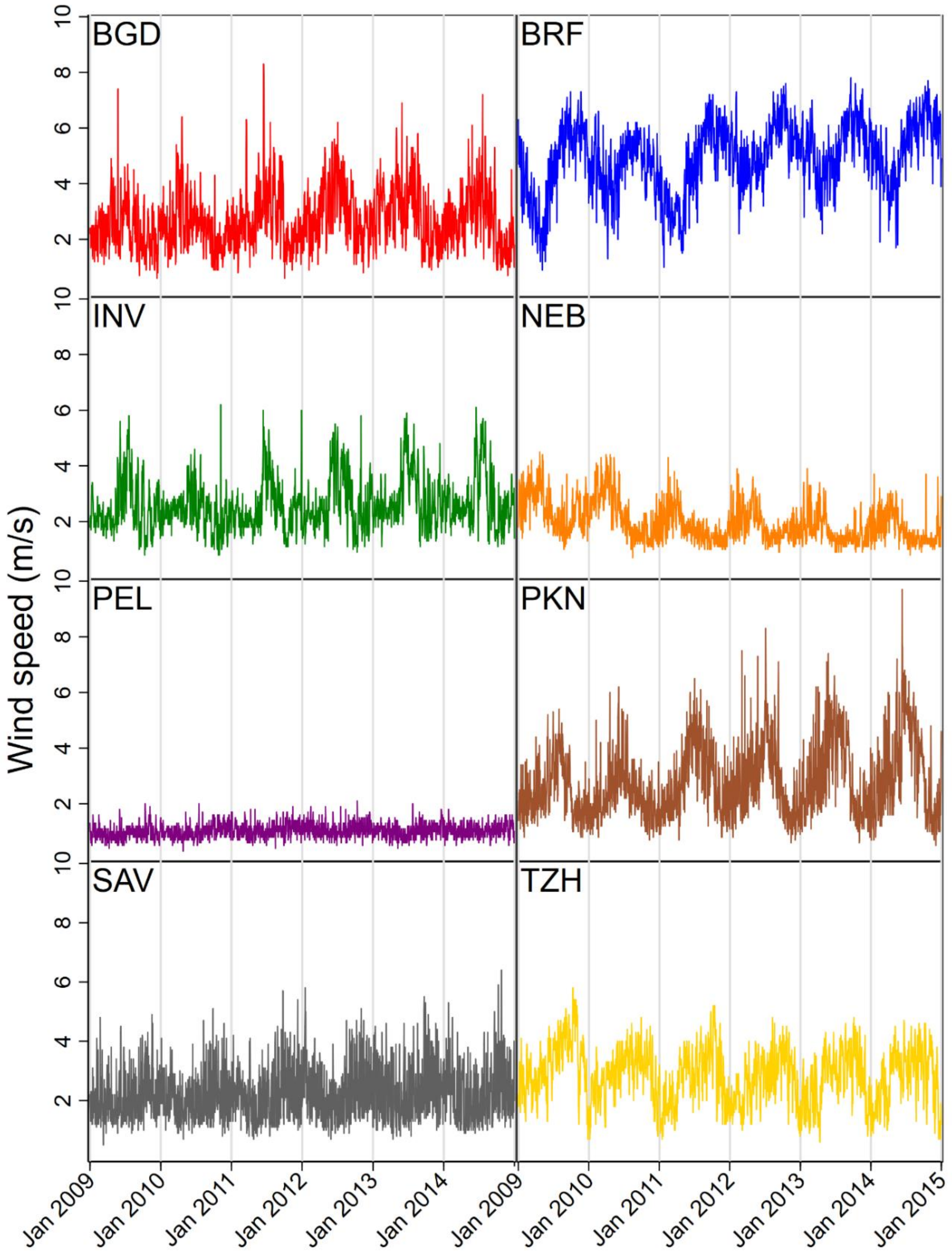

Figure S10: Daily average wind speed estimates from GLDAS at the eight MAL-ED sites, 2009 - 2014[9]

[9] BGD = Dhaka, Bangladesh; BRF = Fortaleza, Brazil; INV = Vellore, India; NEB = Bhaktapur, Nepal; PKN = Naushero Feroze Pakistan; PEL = Loreto, Peru; SAV = Venda, South Africa; TZH = Haydom, Tanzania. Adapted from Colston et al. 2018

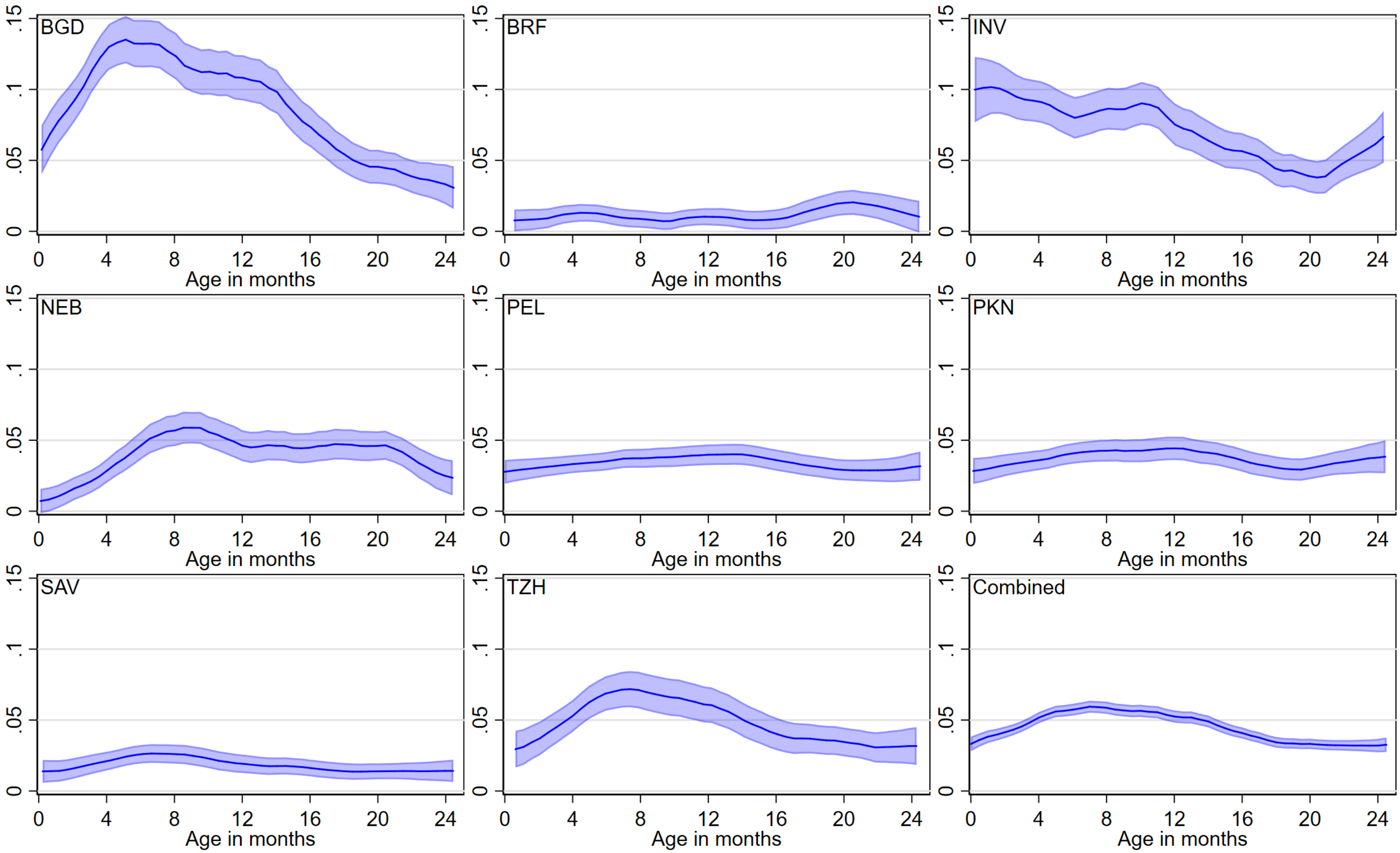

Figure S11: Polynomial smooth plots of the unadjusted association between subjects' age and the probability of contributing a rotavirus-positive stool sample for each MAL-ED site and for all sites combined[10]

[10] BGD = Dhaka, Bangladesh; BRF = Fortaleza, Brazil; INV = Vellore, India; NEB = Bhaktapur, Nepal; PKN = Naushero Feroze Pakistan; PEL = Loreto, Peru; SAV = Venda, South Africa; TZH = Haydom, Tanzania

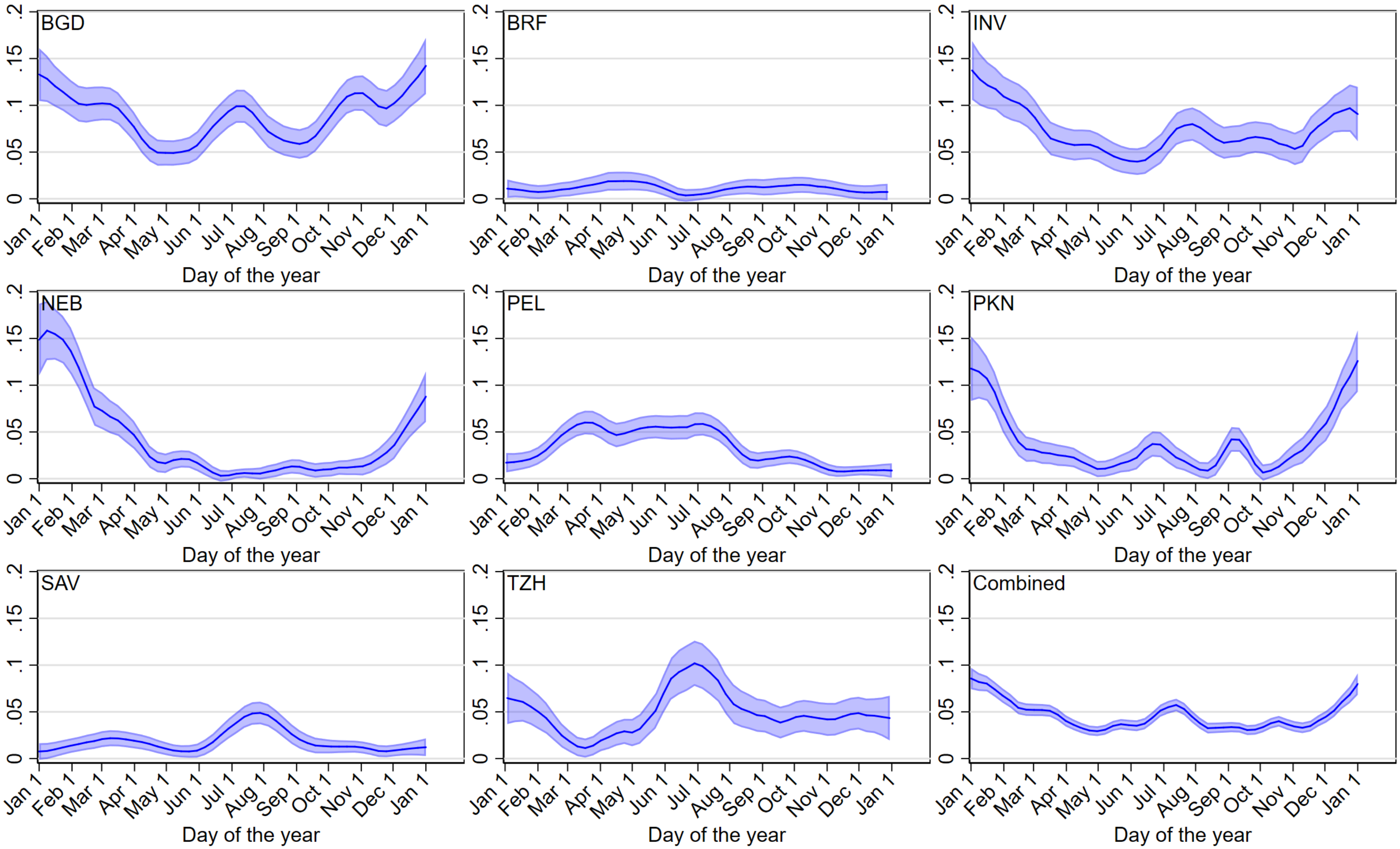

Figure S2: Polynomial smooth plots of the unadjusted association between the day of the year of sample collection and the probability of the sample testing positive for rotavirus for each MAL-ED site and for all sites combined[11]

[11] BGD = Dhaka, Bangladesh; BRF = Fortaleza, Brazil; INV = Vellore, India; NEB = Bhaktapur, Nepal; PKN = Naushero Feroze Pakistan; PEL = Loreto, Peru; SAV = Venda, South Africa; TZH = Haydom, Tanzania

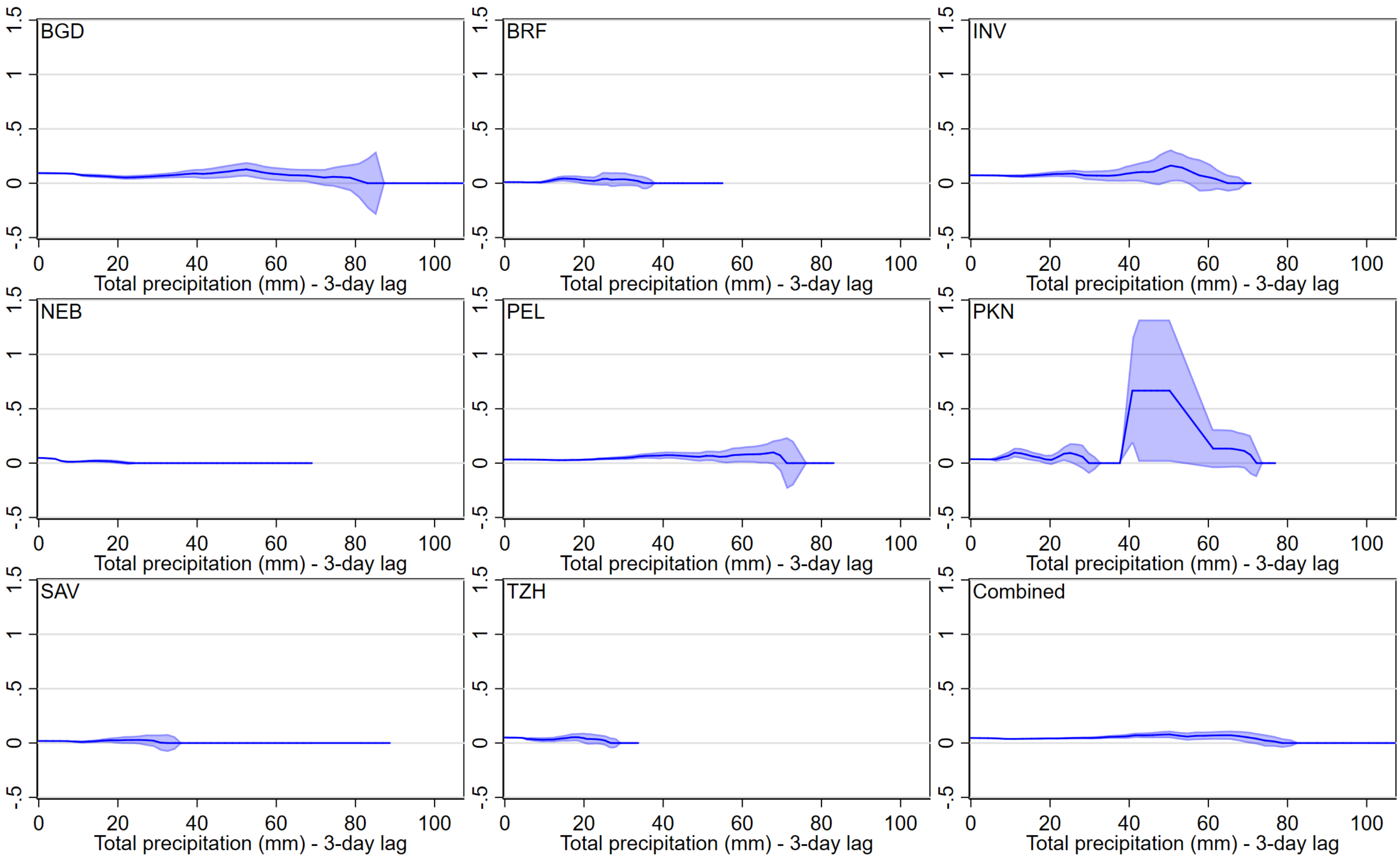

Figure S13: Polynomial smooth plots of the unadjusted association between total daily precipitation and the probability of contributing a rotavirus-positive stool sample (lagged by three days) for each MAL-ED site and for all sites combined[12]

[12] BGD = Dhaka, Bangladesh; BRF = Fortaleza, Brazil; INV = Vellore, India; NEB = Bhaktapur, Nepal; PKN = Naushero Feroze Pakistan; PEL = Loreto, Peru; SAV = Venda, South Africa; TZH = Haydom, Tanzania

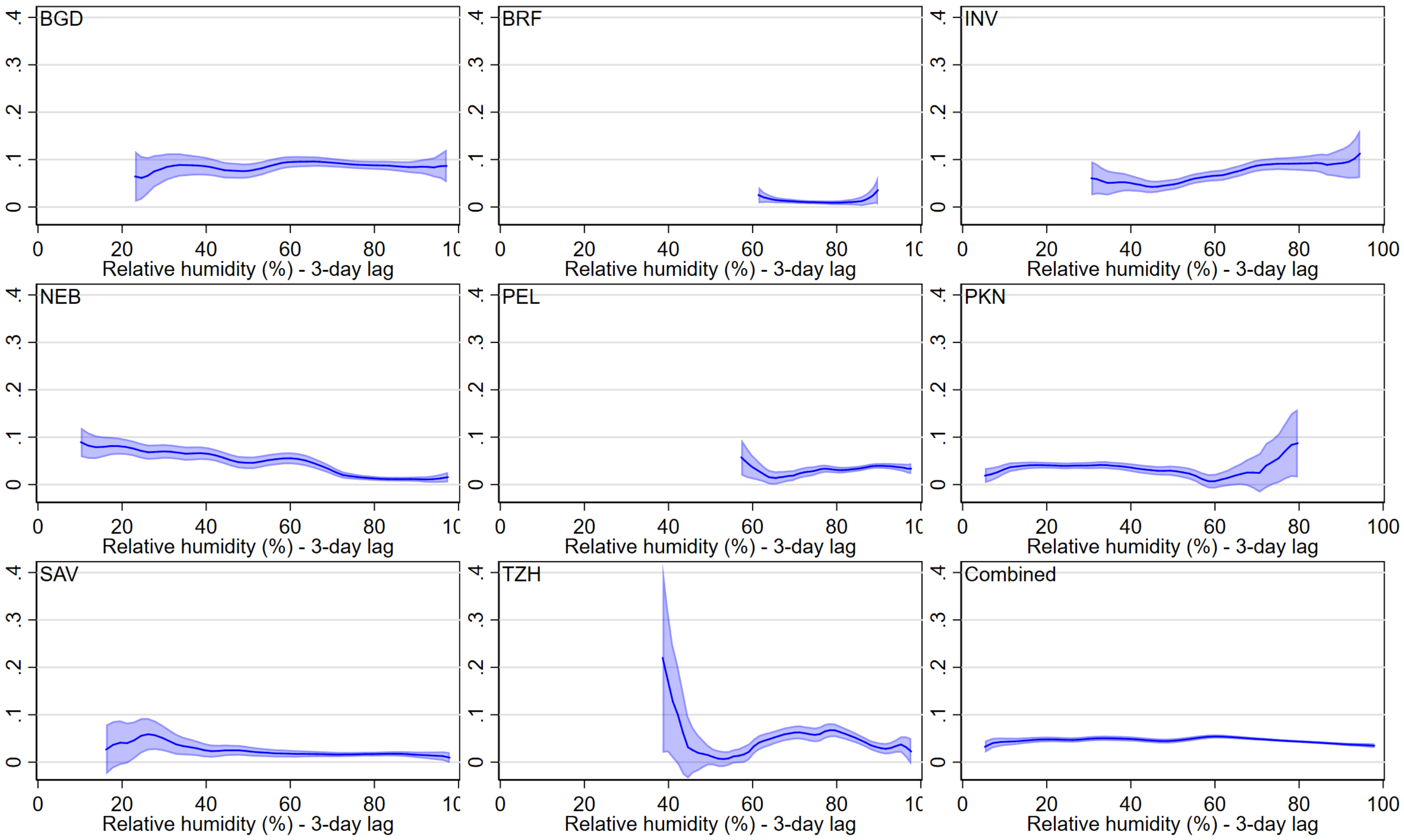

Figure S14: Polynomial smooth plots of the unadjusted association between average daily relative humidity and the probability of contributing a rotavirus-positive stool sample (lagged by three days) for each MAL-ED site and for all sites combined[13]

[13] BGD = Dhaka, Bangladesh; BRF = Fortaleza, Brazil; INV = Vellore, India; NEB = Bhaktapur, Nepal; PKN = Naushero Feroze Pakistan; PEL = Loreto, Peru; SAV = Venda, South Africa; TZH = Haydom, Tanzania

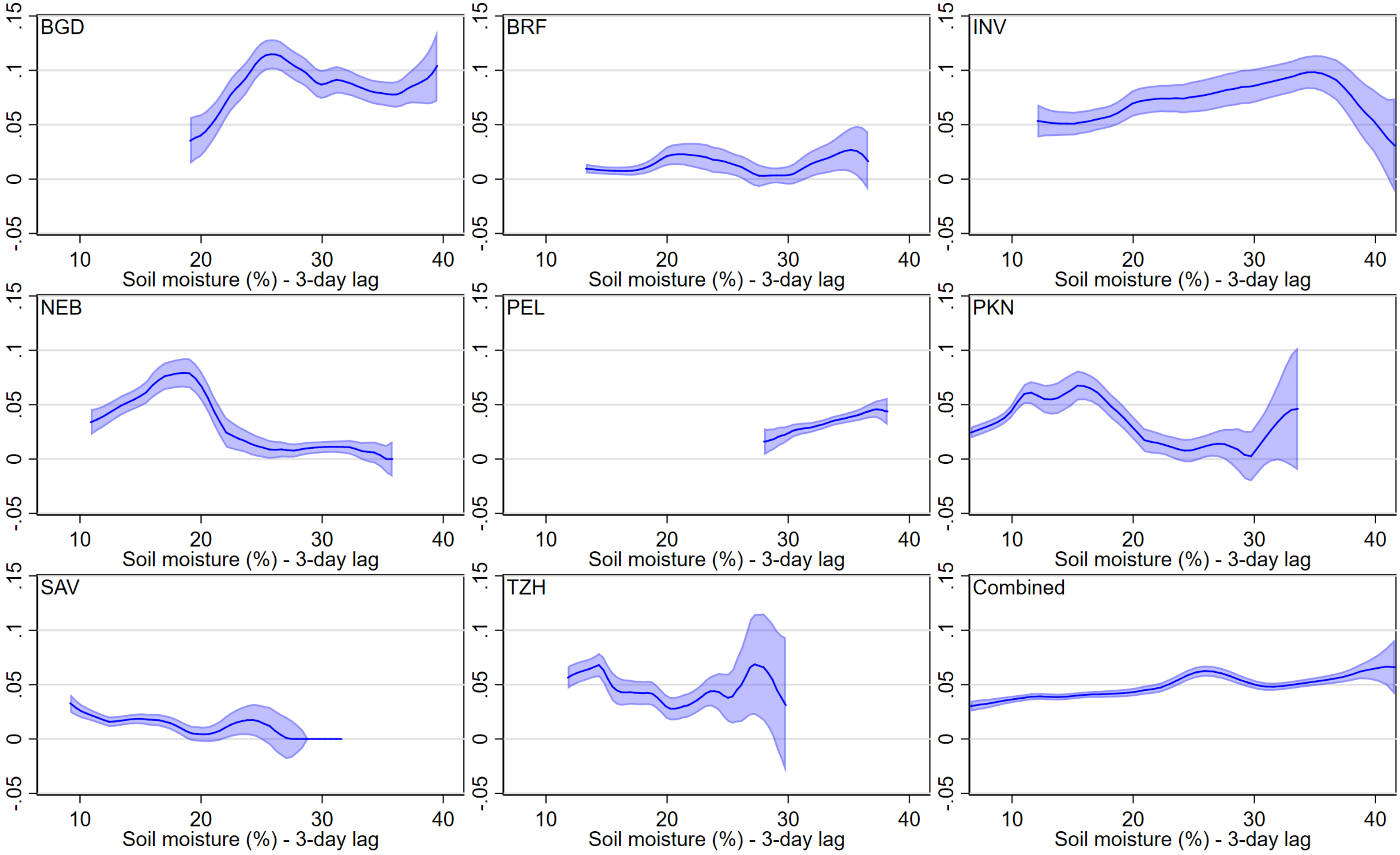

Figure S15: Polynomial smooth plots of the unadjusted association between average daily soil moisture and the probability of contributing a rotavirus-positive stool sample (lagged by three days) for each MAL-ED site and for all sites combined[14]

[14] BGD = Dhaka, Bangladesh; BRF = Fortaleza, Brazil; INV = Vellore, India; NEB = Bhaktapur, Nepal; PKN = Naushero Feroze Pakistan; PEL = Loreto, Peru; SAV = Venda, South Africa; TZH = Haydom, Tanzania

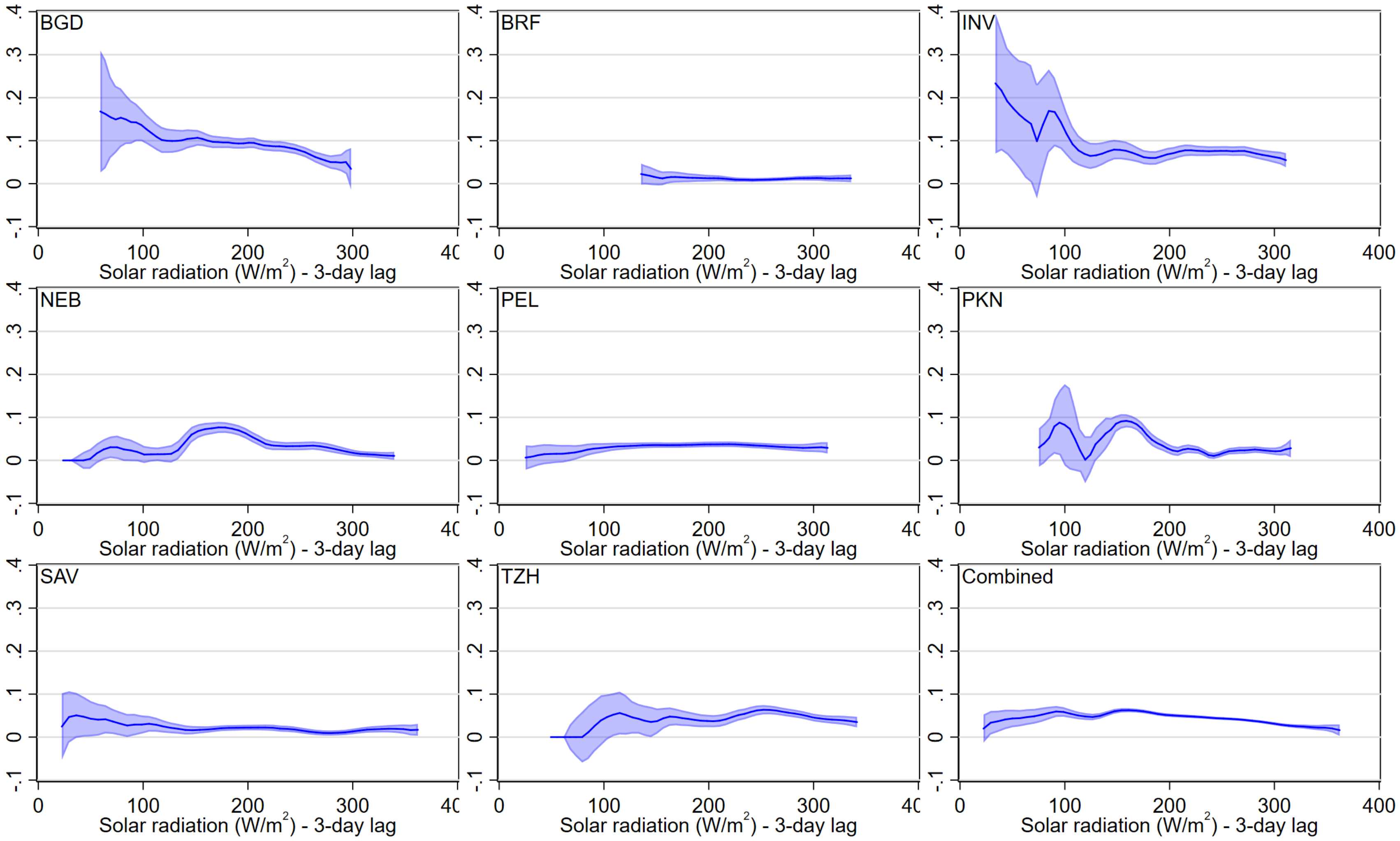

Figure S16: Polynomial smooth plots of the unadjusted association between average daily solar radiation and the probability of contributing a rotavirus-positive stool sample (lagged by three days) for each MAL-ED site and for all sites combined[15]

[15] BGD = Dhaka, Bangladesh; BRF = Fortaleza, Brazil; INV = Vellore, India; NEB = Bhaktapur, Nepal; PKN = Naushero Feroze Pakistan; PEL = Loreto, Peru; SAV = Venda, South Africa; TZH = Haydom, Tanzania

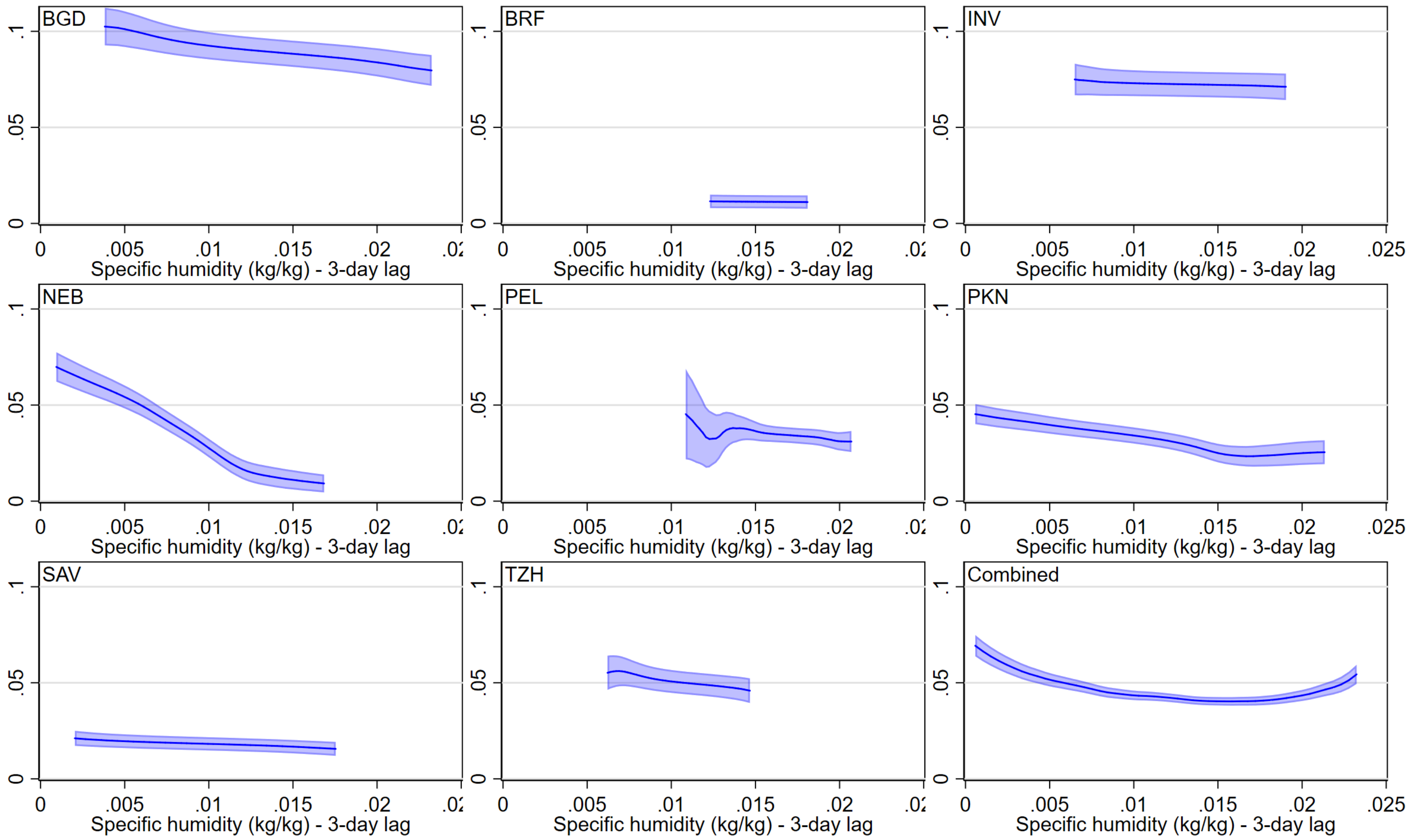

Figure S17: Polynomial smooth plots of the unadjusted association between average daily specific humidity and the probability of contributing a rotavirus-positive stool sample (lagged by three days) for each MAL-ED site and for all sites combined[16]

[16] BGD = Dhaka, Bangladesh; BRF = Fortaleza, Brazil; INV = Vellore, India; NEB = Bhaktapur, Nepal; PKN = Naushero Feroze Pakistan; PEL = Loreto, Peru; SAV = Venda, South Africa; TZH = Haydom, Tanzania

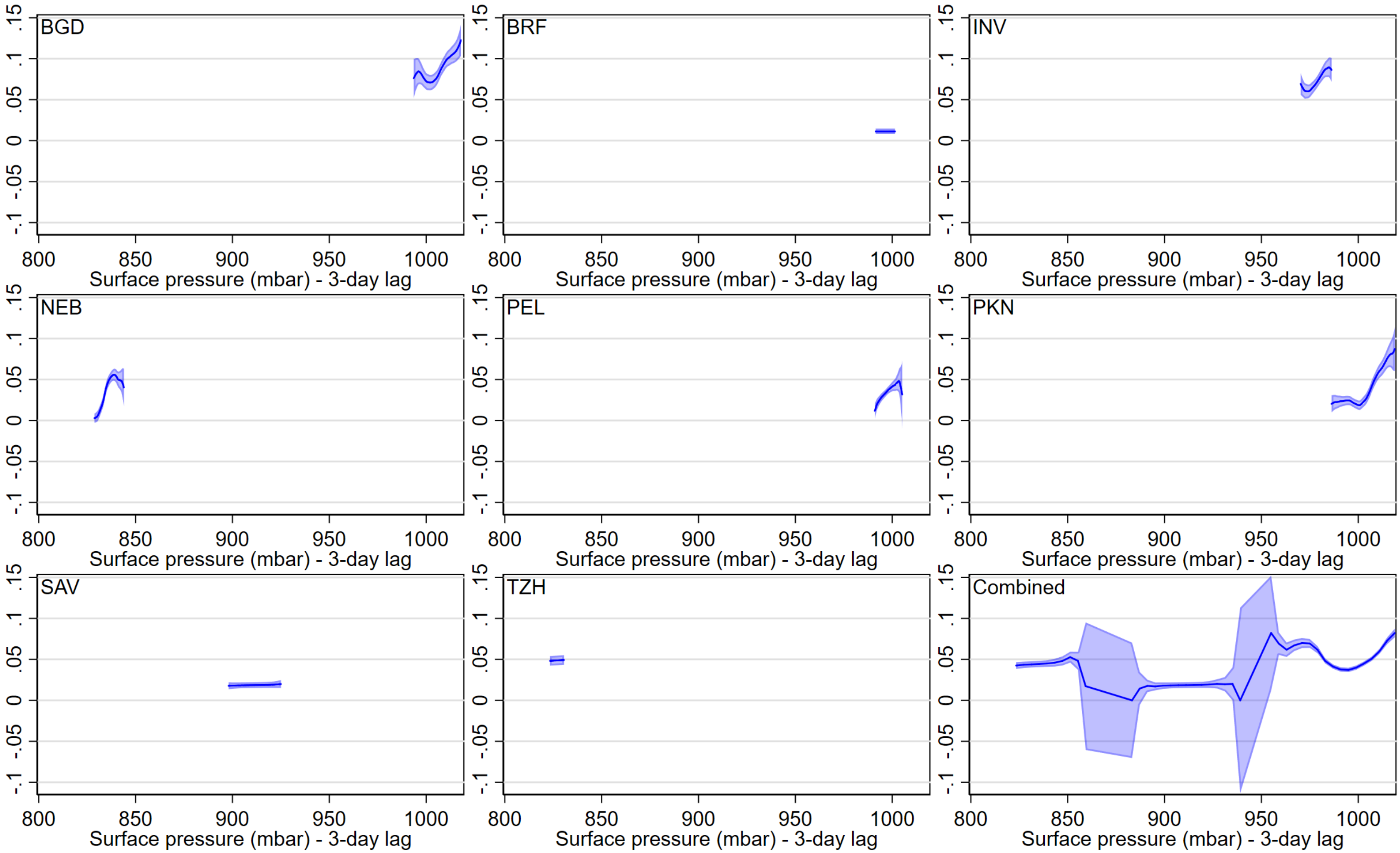

Figure S18: Polynomial smooth plots of the unadjusted association between average daily surface pressure and the probability of contributing a rotavirus-positive stool sample (lagged by three days) for each MAL-ED site and for all sites combined[17]

[17] BGD = Dhaka, Bangladesh; BRF = Fortaleza, Brazil; INV = Vellore, India; NEB = Bhaktapur, Nepal; PKN = Naushero Feroze Pakistan; PEL = Loreto, Peru; SAV = Venda, South Africa; TZH = Haydom, Tanzania

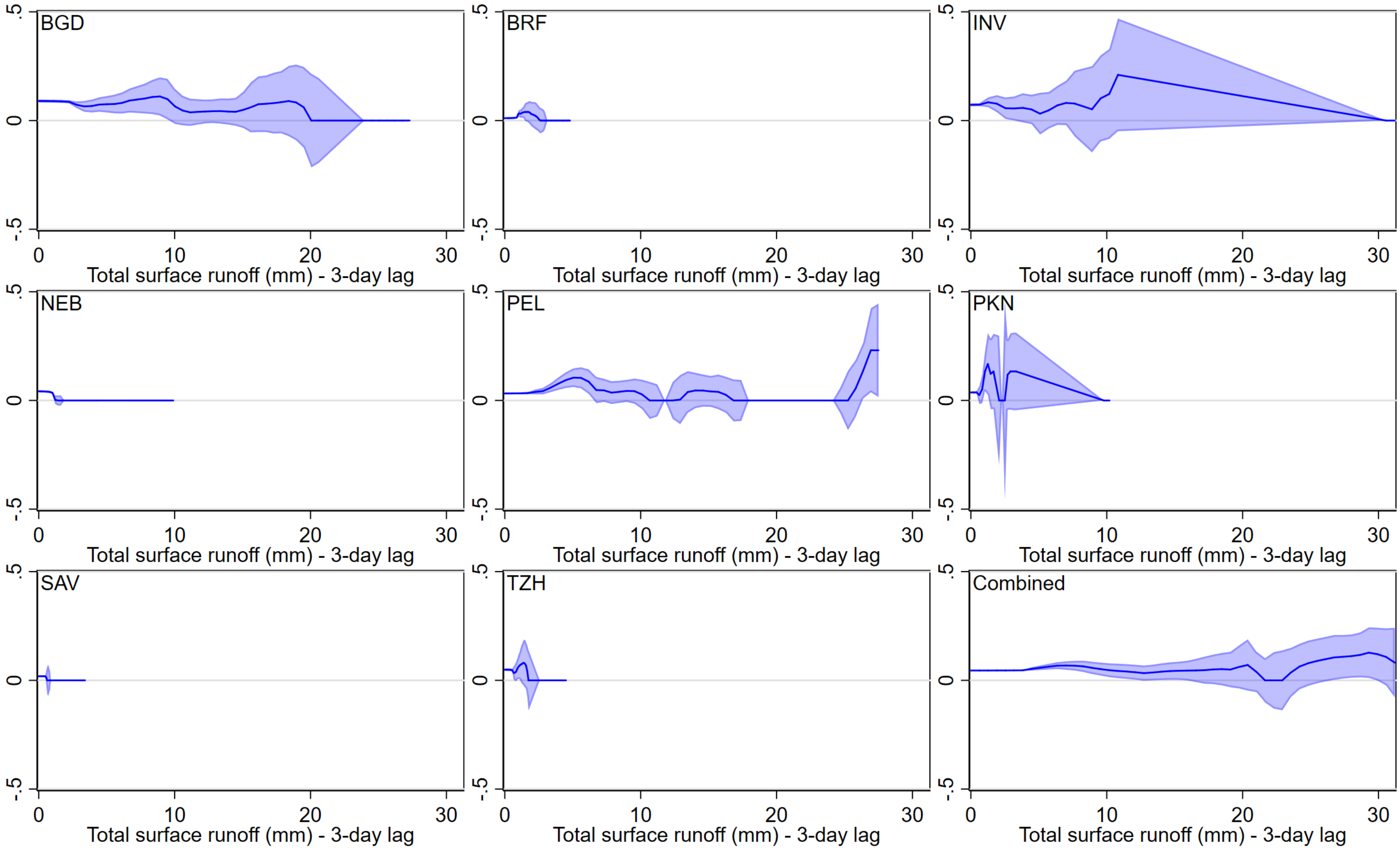

Figure S19: Polynomial smooth plots of the unadjusted association between total daily surface runoff and the probability of contributing a rotavirus-positive stool sample (lagged by three days) for each MAL-ED site and for all sites combined[18]

[18] BGD = Dhaka, Bangladesh; BRF = Fortaleza, Brazil; INV = Vellore, India; NEB = Bhaktapur, Nepal; PKN = Naushero Feroze Pakistan; PEL = Loreto, Peru; SAV = Venda, South Africa; TZH = Haydom, Tanzania

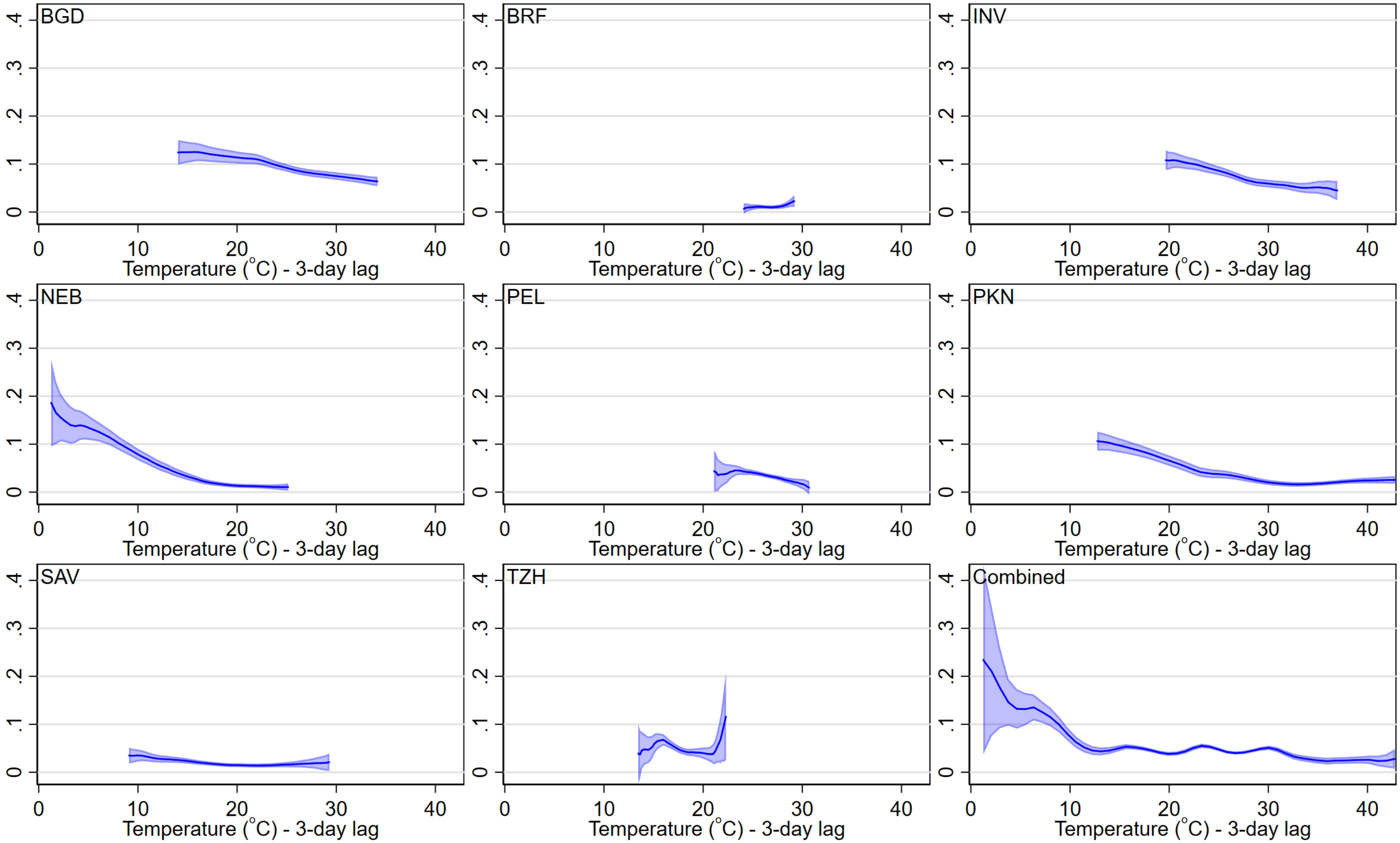

Figure S20: Polynomial smooth plots of the unadjusted association between average daily temperature and the probability of contributing a rotavirus-positive stool sample (lagged by three days) for each MAL-ED site and for all sites combined[19]

[19] BGD = Dhaka, Bangladesh; BRF = Fortaleza, Brazil; INV = Vellore, India; NEB = Bhaktapur, Nepal; PKN = Naushero Feroze Pakistan; PEL = Loreto, Peru; SAV = Venda, South Africa; TZH = Haydom, Tanzania

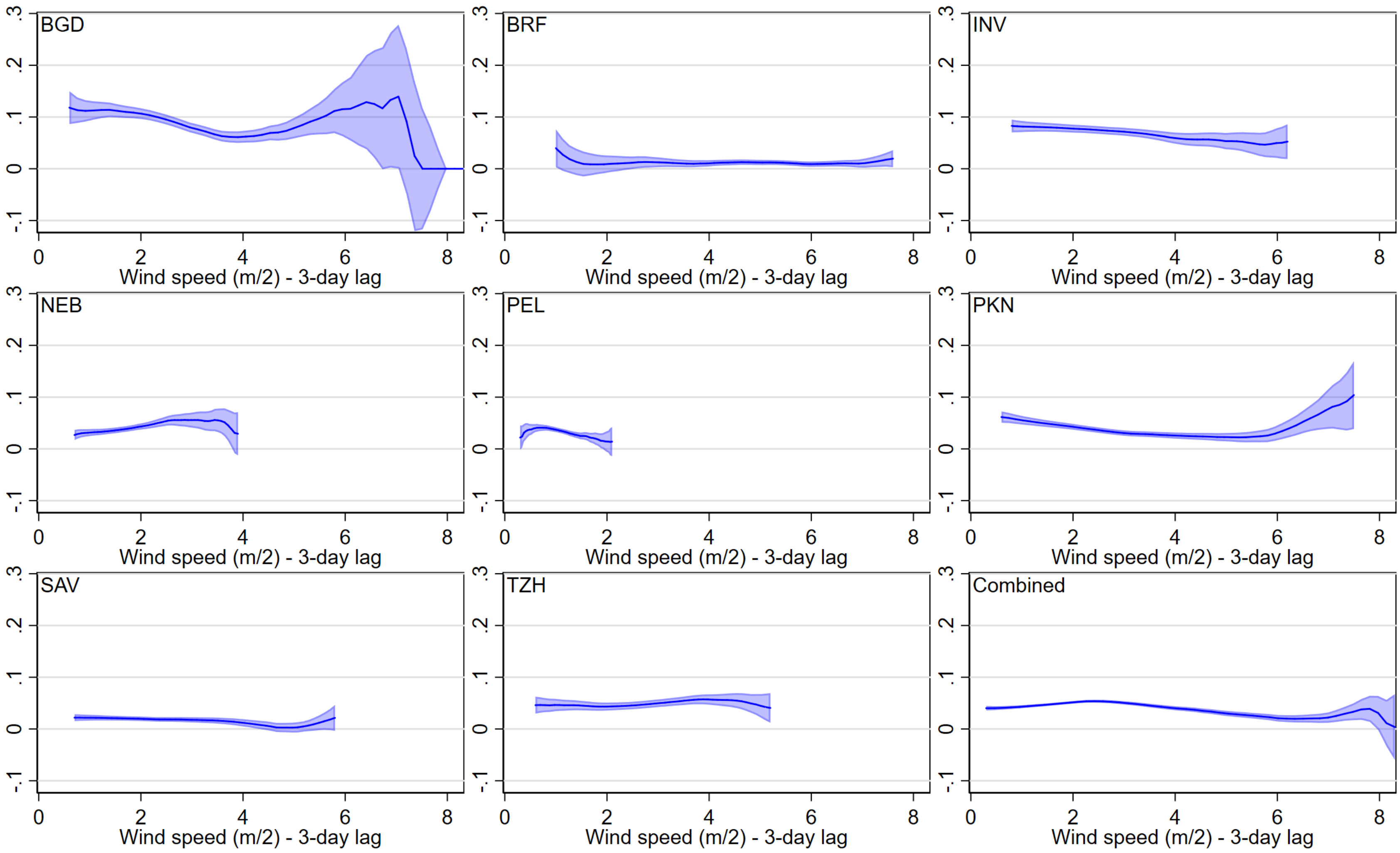

Figure S21: Polynomial smooth plots of the unadjusted association between average daily wind speed and the probability of contributing a rotavirus-positive stool sample (lagged by three days) for each MAL-ED site and for all sites combined[20]

[20] BGD = Dhaka, Bangladesh; BRF = Fortaleza, Brazil; INV = Vellore, India; NEB = Bhaktapur, Nepal; PKN = Naushero Feroze Pakistan; PEL = Loreto, Peru; SAV = Venda, South Africa; TZH = Haydom, Tanzania

Lag analysis:

To determine the optimal lag lengths for subsequent analysis, exploratory models were fitted separately for each lag length from 2 to 10 days in turn ("individual lag models" - ILMs) and compared with distributed lag models (DLMs) incorporating all terms for all considered lag lengths. This window of plausible lag lengths was bounded by the shortest estimated rotavirus incubation period (1·4 days[1]) and the longest documented serial interval (9 days[2]) plus an additional day for reporting symptoms.[3] For each variable and effect, lag lengths that were significant at the α =0·05 level in both the ILM and DLM were retained in further models. If no lag length met this criterion, the single length with the lowest average of the two p-values was selected.

Five variables –Relative humidity, soil moisture, specific humidity, surface pressure and temperature – were highly statistically significantly associated with rotavirus infection for all (or almost all) lag lengths and both effects. For two other variables this was true for the absolute effect, but the adjusted effect either varied from non-significant to highly significant (solar radiation) or was only moderately significant across one lag length (wind speed). Conversely, the adjusted effect of precipitation was highly statistically significant for almost every lag, while the absolute effect was only slightly statistically significant for 8 days. Surface runoff showed a similar pattern to precipitation but was slightly statistically significant at 3- and 9-day lags, and moderately so at 8 days. Notably, two pairs of consecutive lag lengths of wind speed – 2-, 3- 8- and 9-day lags – were significant in the absolute effect DLM and retained by stepwise selection.

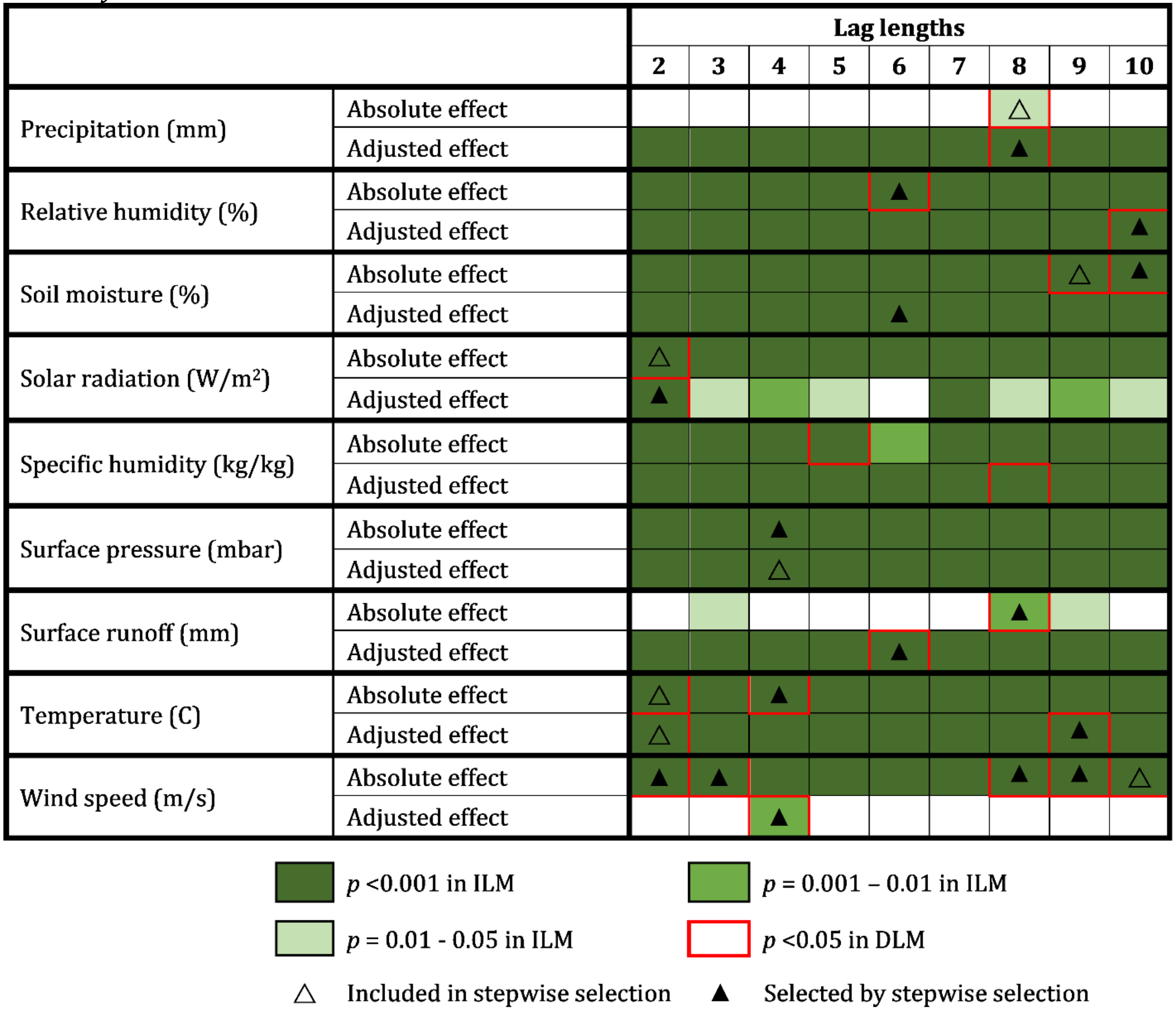

Figure S22: Significance levels of associations found by individual (ILM) and distributed lag models (DLM) and selected lag lengths for effects of nine hydrometeorological variables on rotavirus infection status.[21]

[21] Specific humidity was excluded from the multi-variable models.

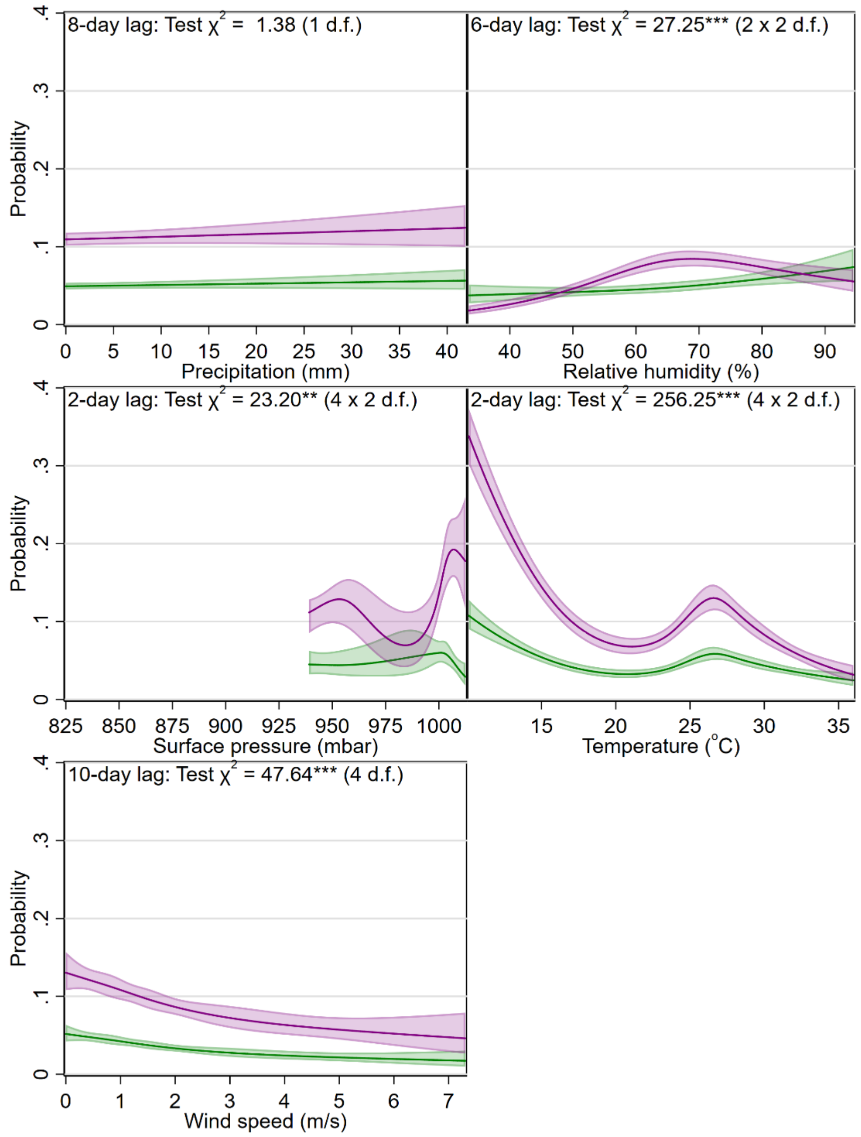

Figure S23: Probabilities of rotavirus infection predicted by single-variable, absolute effect models for hydrometeorological variables measured at weather stations nearby to those MAL-ED sites for which such data was available, for both episode types - symptomatic (shown in purple, the probability of rotavirus-positivity for diarrheal stool) and asymptomatic (shown in green, the probability for a non-diarrheal stool).[22] Weather station data on precipitation was available for all sites, temperature for all except TZH, surface pressure and wind speed for all except INV and TZH and relative humidity available only for INV, PKN and SAV. Distances from sites to their nearest weather station varied from 1km to 37km.

[22] "d.f." = degrees of freedom. Variables with "x 2 d.f." are those for which terms for an interaction with sample type were included. *** p<0.001, ** p=0.001 – 0.01, * p=0.01 – 0.05. For variables for which multiple lags met the criteria for inclusion in subsequent models, only the one with the highest level of statistical significance is shown, but the magnitude and shape of the association did not change substantially for the other lag lengths (not shown).

Table S1: Absolute effect – risk ratios, 95% confidence intervals (CI) and p-values from the final main effect and interaction Poisson models fitted with generalized linear models using absolute values for the exposures before adjusting for seasonality[23]

	Main effects model		Interaction model	
	**Risk ratio (95% CI)**	**p-value**	**Risk ratio (95% CI)**	**p-value**
**Baseline rate**	90.86 (0.20, 412x10^{4})	0.15	1.92x10^{3} (0.00, 2.22x10^{31})	0.82
**Vaccine category**	0.43 (0.35, 0.52)	<0.0001	9.42x10^{28} (0.00, 2.00x10^{133})	0.59
**Age**		**<0.0001**		**<0.0001**
Linear	1.25 (1.17, 1.33)	<0.0001	1.25 (1.17, 1.34)	<0.0001
Quadratic	0.98 (0.98, 0.99)	<0.0001	0.98 (0.97, 0.99)	<0.0001
Cubic	1.00 (1.00, 1.00)	<0.0001	1.00 (1.00, 1.00)	<0.0001
**Sample type**	2.65 (2.42, 2.91)	<0.0001	0.00 (0.00, 46.80)	0.13
**Relative humidity 6-day lag**		**<0.0001**		**<0.01**
Spline 1	1.01 (1.01, 1.02)	<0.0001	1.02 (0.99, 1.04)	0.16
Spline 2	0.99 (0.98, 0.99)	<0.001	0.95 (0.92, 0.99)	<0.01
**Soil moisture 10-day lag**		**<0.001**		**0.15**
Spline 1	0.99 (0.97, 1.00)	0.08	7.84 (0.97, 63.29)	0.05
Spline 2	1.03 (1.01, 1.05)	<0.001	0.01 (0.00, 6.52)	0.17
**Surface pressure 4-day lag**		**<0.0001**		**0.49**
Spline 1	0.99 (0.99, 1.00)	0.08	0.99 (0.92, 1.07)	0.76
Spline 2	1.04 (1.02, 1.05)	<0.0001	1.09 (0.89, 1.32)	0.41
Spline 3	0.84 (0.79, 0.90)	<0.0001	0.66 (0.28, 1.59)	0.36
Spline 4	2.43x10^{3} (41.43, 1.43x10^{5})	<0.001	6.18x10^{3} (0.00, 2.08x10^{22})	0.69
**Surface runoff 8-day lag**	1.03 (1.01, 1.05)	0.01	-	-
**Temperature 4-day lag**		**<0.01**		**0.39**
Spline 1	0.87 (0.85, 0.88)	<0.0001	0.93 (0.77, 1.12)	0.42
Spline 2	1.45 (1.25, 1.68)	<0.0001	1.06 (3.43, 3.27)	0.92
Spline 3	0.27 (0.15, 0.50)	<0.0001	0.58 (0.01, 56.60)	0.82
Spline 4	11.19 (2.51, 49.89)	<0.001	14.10 (0.00, 5.13x10^{5})	0.62
**Wind speed 2-day lag**		**<0.01**		**<0.001**
Spline 1	078 (0.52, 1.16)	0.22	0.53 (0.18, 1.54)	0.25
Spline 2	637.79 (3.09, 1.32x10^{5})	0.02	6.51x10^{3} (0.00, 1.19x10^{12})	0.37
Spline 3	0.00 (0.00, 0.05)	0.02	0.00 (0.00, 1.34x1013)	0.46
Spline 4	1.39x10^{5} (5.59, 3.46x10^{9})	0.02	3.04x10^{6} (0.00, 1.56x10^{26})	0.52
**Wind speed 3-day lag**		**0.05**		**0.23**
Spline 1	0.59 (0.40, 0.86)	0.01	0.00 (0.00, 7.56x10^{13})	0.67
Spline 2	263.40 (1.84, 3.78x10^{4})	0.03	3.01x10^{48} (0.00, 1.70x10^{272})	0.67
Spline 3	0.00 (0.00, 0.49)	0.04	0.00 (0.00, .)	0.76
Spline 4	9.86x10^{3} (0.65, 1.50x10^{8})	0.06	5.81x10^{31} (0.00, .)	0.89
**Wind speed 8-day lag**		**0.02**		**0.46**
Spline 1	1.07 (0.92, 1.23)	0.39	0.55 (0.21, 1.41)	0.21
Spline 2	0.80 (0.64, 1.00)	0.05	3.06 (0.29, 31.87)	0.35
**Wind speed 9-day lag**		**<0.001**		-
Spline 1	0.70 (0.48, 1.02)	0.06	-	-
Spline 2	2.06x10^{3} (14.41, 2.94x10^{5})	<0.001	-	-
Spline 3	0.00 (0.00, 0.00)	<0.001	-	-
Spline 4	1.06x10^{6} (102.93, 1.10x10^{10})	<0.001	-	-
**Vaccine/age interaction**		-		**0.11**
Linear	-	-	0.90 (0.16, 1.06)	0.21
Quadratic	-	-	1.01 (0.99, 1.03)	0.21
Cubic	-	-	1.00 (1.00, 1.00)	0.28

[23] Some parameters are omitted because they were either to large to calculate or were excluded due to collinearity.

Table S1: Absolute effect – risk ratios, 95% confidence intervals (CI) and p-values from the final main effect and interaction Poisson models fitted with generalized linear models using absolute values for the exposures before adjusting for seasonality[23]

	Main effects model		Interaction model	
	**Risk ratio (95% CI)**	**p-value**	**Risk ratio (95% CI)**	**p-value**
**Vaccine/surface pressure interaction**		-		**<0.0001**
Spline 1	-	-	0.93 (0.71, 1.22)	0.62
Spline 2	-	-	0.93 (0.66, 1.31)	0.67
Spline 3	-	-	1.93 (0.67, 5.56)	0.22
Spline 4	-	-	0.00 (0.00, 3.18x10^{5})	0.12
**Vaccine/temperature interaction**		-		<0.001
Spline 1	-	-	0.84 (0.75, 0.95)	<0.01
Spline 2	-	-	4.61 (1.20, 9.67)	<0.001
Spline 3	-	-	0.00 (0.00, 0.10)	<0.01
Spline 4	-	-	5.96x10^{3} (0.08, 4.57x10^{8})	0.13
**Sample type/soil moisture interaction**				**<0.0001**
Spline 1	-	-	1.05 (1.02, 1.09)	<0.01
Spline 2	-	-	0.97 (0.92, 1.02)	0.28
**Sample type/surface pressure interaction**		-		
Spline 1	-	-	1.01 (0.99, 1.03)	0.16
Spline 2	-	-	0.95 (0.91, 0.99)	0.02
Spline 3	-	-	1.28 (1.09, 1.49)	<0.01
Spline 4	-	-	0.00 (0.00, 2.54)	0.09
**Sample type/wind speed (3-day lag) interaction**		-		**<0.0001**
Spline 1	-	-	2.30 (1.14, 4.67)	0.02
Spline 2	-	-	0.00 (0.00, 4.79)	0.10
Spline 3	-	-	6.52x10^{7} (0.01, 5.77x10^{17})	0.12
Spline 4	-	-	0.00 (0.00, 506.28)	0.19
**Temperature/relative humidity interaction**		-		**<0.0001**
Spline 1 * spline 1	-	-	1.00 (1.00, 1.00)	0.94
Spline 1 * spline 2	-	-	1.00 (1.00, 1.00)	0.05
Spline 2 * spline 1	-	-	0.98 (0.96, 0.99)	<0.01
Spline 2 * spline 2	-	-	1.01 (0.99, 1.02)	0.31
Spline 3 * spline 1	-	-	1.15 (1.07, 1.24)	<0.001
Spline 3 * spline 2	-	-	0.92 (0.86, 0.99)	0.04
Spline 4 * spline 1	-	-	0.69 (0.56, 0.84)	<0.001
Spline 4 * spline 2	-	-	1.35 (1.09, 1.67)	<0.01
**Temperature/soil moisture interaction**		-		**<0.0001**
Spline 1 * spline 1	-	-	0.99 (0.98, 1.00)	0.23
Spline 1 * spline 2	-	-	1.03 (1.00, 1.07)	0.05
Spline 2 * spline 1	-	-	1.07 (1.00, 1.15)	0.05
Spline 2 * spline 2	-	-	0.92 (0.80, 1.04)	0.18
Spline 3 * spline 1	-	-	0.70 (0.51, 0.95)	0.02
Spline 3 * spline 2	-	-	1.14 (0.88, 2.28)	0.16
Spline 4 * spline 1	-	-	2.37 (1.10, 5.12)	0.03
Spline 4 * spline 2	-	-	0.45 (0.15, 1.35)	0.16

Table S1: Absolute effect – risk ratios, 95% confidence intervals (CI) and p-values from the final main effect and interaction Poisson models fitted with generalized linear models using absolute values for the exposures before adjusting for seasonality[23]

	Main effects model		Interaction model	
	**Risk ratio (95% CI)**	**p-value**	**Risk ratio (95% CI)**	**p-value**
**Soil moisture/surface pressure interaction**		-		**<0.0001**
Spline 1 * spline 1	-	-	1.00 (1.00, 1.00)	0.06
Spline 1 * spline 2	-	-	1.01 (1.00, 1.01)	0.04
Spline 2 * spline 1	-	-	0.98 (0.96, 1.00)	0.10
Spline 2 * spline 2	-	-	1.53 (0.39, 6.01)	0.54
Spline 3 * spline 1	-	-	1.00 (1.00, 1.01)	0.23
Spline 3 * spline 2	-	-	0.99 (0.97, 1.00)	0.14
Spline 4 * spline 1	-	-	1.04 (0.99, 1.10)	0.12
Spline 4 * spline 2	-	-	0.46 (0.06, 3.29)	0.44
**Wind speed (3-day lag)/surface pressure interaction**		-		**<0.0001**
Spline 1 * spline 1	-	-	1.01 (0.96, 1.06)	0.65
Spline 1 * spline 2	-	-	0.96 (0.85, 1.08)	0.49
Spline 1 * spline 3	-	-	1.20 (0.68, 2.12)	0.53
Spline 1 * spline 4	-	-	0.00 (0.00, 2.72×10^{9})	0.64
Spline 2 * spline 1	-	-	0.87 (0.47, 1.61)	0.67
Spline 2 * spline 2	-	-	1.59 (0.34, 7.51)	0.56
Spline 2 * spline 3	-	-	0.11 (0.00, 112.57)	0.53
Spline 2 * spline 4	-	-	2.99×10^{78} (0.00, 2.1×10^{235})	0.33
Spline 3 * spline 1	-	-	1.26 (0.30, 5.40)	0.75
Spline 3 * spline 2	-	-	0.42 (0.01, 15.50)	0.64
Spline 3 * spline 3	-	-	95.07 (0.00, 8.20×10^{8})	0.58
Spline 3 * spline 4	-	-	0.00 (0.00, 1.20×10^{154})	0.26
Spline 4 * spline 1	-	-	0.91 (0.25, 3.29)	0.89
Spline 4 * spline 2	-	-	1.56 (0.73, 33.27)	0.78
Spline 4 * spline 3	-	-	0.06 (0.00, 2.43×10^{4})	0.68
Spline 4 * spline 4	-	-	7.00×10^{191} (0.00, .)	0.20
**Wind speed (2-day lag)/wind speed (8-day lag)**		-		**<0.0001**
Spline 1 * spline 1	-	-	1.73 (0.79, 3.76)	0.17
Spline 1 * spline 2	-	-	0.32 (0.05, 2.08)	0.23
Spline 2 * spline 1	-	-	0.00 (0.00, 232.37)	0.28
Spline 2 * spline 2	-	-	1.22×10^{8} (0.00, 1.32×10^{19})	0.15
Spline 3 * spline 1	-	-	1.16×10^{6} (0.00, 6.68×10^{18})	0.35
Spline 3 * spline 2	-	-	0.00 (0.00, 2.19×10^{6})	0.14
Spline 4 * spline 1	-	-	0.00 (0.00, 3.10×10^{6})	0.42
Spline 4 * spline 2	-	-	3.61×10^{15} (0.00, 2.03×10^{35})	0.12

Table S2: Adjusted effect – risk ratios, 95% confidence intervals (CI) and p-values from the final main effect and interaction Poisson models fitted with generalized linear models using deviations of the exposures from the site-specific average adjusting for seasonality. [24]

	Main effects model		Interaction model	
	**Risk ratio (95%CI)**	**p-value**	**Risk ratio**	**p-value**
**Baseline rate**	0.20 (0.06, 0.64)	<0.01	21.58 (0.35, 1.32x10^{3})	0.14
**Vaccine category**	0.35 (0.29, 0.42)	<0.0001	0.02 (0.00, 0.23)	<0.01
**Age**		**<0.0001**		**<0.0001**
Linear	1.25 (1.18, 1.33)	<0.0001	1.28 (1.19, 1.36)	<0.0001
Quadratic	0.98 (0.97, 0.99)	<0.0001	0.98 (0.97, 0.98)	<0.0001
Cubic	0.35 (0.29, 0.42)	<0.0001	1.00 (1.00, 1.00)	<0.0001
**Sample type**	2.61 (2.38, 2.85)	<0.0001	0.34 (0.10, 1.14)	0.08
**Seasonality**		**<0.0001**		**<0.0001**
BGD * 1st sine function	0.97 (0.82, 1.15)	0.73	1.06 (0.87, 1.30)	0.57
BRF * 1st sine function	0.86 (0.63, 1.18)	0.36	1.07 (0.71, 1.62)	0.75
INV * 1st sine function	1.03 (0.82, 1.29)	0.79	1.14 (0.91, 1.43)	0.24
NEB * 1st sine function	1.35 (1.05, 1.74)	0.02	1.32 (0.97, 1.81)	0.08
PEL * 1st sine function	1.61 (1.30, 1.99)	<0.0001	1.74 (1.34, 2.26)	<0.0001
PKN * 1st sine function	1.01 (0.81, 1.26)	0.92	0.93 (0.71, 1.22)	0.60
SAV * 1st sine function	1.20 (0.73, 1.98)	0.47	1.39 (0.95, 2.03)	0.09
TZH * 1st sine function	0.81 (0.64, 1.04)	0.09	0.64 (0.49, 0.84)	<0.01
BGD * 2nd sine function	0.97 (0.86, 1.10)	0.67	1.00 (0.87, 1.15)	0.97
BRF * 2nd sine function	0.91 (0.65, 1.27)	0.59	0.95 (0.61, 1.50)	0.84
INV * 2nd sine function	1.23 (1.04, 1.46)	0.02	1.20 (1.02, 1.45)	0.03
NEB * 2nd sine function	1.04 (0.87, 1.24)	<0.01	1.70 (1.34, 2.16)	<0.0001
PEL * 2nd sine function	1.23 (1.05, 1.45)	0.01	1.32 (1.12, 1.56)	<0.01
PKN * 2nd sine function	1.04 (0.87, 1.24)	0.67	1.26 (1.01, 1.57)	0.04)
SAV * 2nd sine function	2.56 (1.69, 3.86)	<0.0001	2.07 (1.48, 2.89)	<0.0001
TZH * 2nd sine function	0.84 (0.70, 1.02)	0.08	0.89 (0.73, 1.08)	0.24
BGD * 1st cosine function	1.22 (0.97, 1.53)	0.10	1.45 (1.09, 1.94)	0.01
BRF * 1st cosine function	0.89 (0.64, 1.24)	0.49	0.91 (0.56, 1.49)	0.72
INV * 1st cosine function	1.38 (1.09, 1.76)	<0.01	1.32 (1.02, 1.71)	0.04
NEB * 1st cosine function	1.61 (1.24, 2.09)	<0.001	1.71 (1.21, 2.42)	<0.01
PEL * 1st cosine function	0.58 (0.48, 0.69)	<0.0001	0.54 (0.45, 0.66)	<0.0001
PKN * 1st cosine function	0.95 (0.68, 1.34)	0.78	1.03 (0.68, 1.55)	0.91
SAV * 1st cosine function	0.60 (0.39, 0.93)	0.02	0.82 (0.51, 1.34)	0.43
TZH * 1st cosine function	0.74 (0.62, 0.89)	<0.01	0.66 (0.55, 0.81)	<0.0001
BGD * 2nd cosine function	1.38 (1.20, 1.58)	<0.0001	1.54 (1.32, 1.80)	<0.0001
BRF * 2nd cosine function	0.72 (0.52, 0.99)	0.04	0.56 (0.36, 0.86)	<0.01
INV * 2nd cosine function	1.41 (1.18, 1.69)	<0.001	1.54 (1.37, 1.86)	<0.0001
NEB * 2nd cosine function	1.67 (1.40, 1.99)	<0.0001	2.03 (1.62, 2.54)	<0.0001
PEL * 2nd cosine function	0.92 (0.77, 1.10)	0.35	0.88 (0.73, 1.06)	0.19
PKN * 2nd cosine function	1.99 (1.60, 2.47)	<0.0001	2.13 (1.66, 2.74)	<0.0001
SAV * 2nd cosine function	1.33 (0.90, 1.95)	0.15	1.12 (0.81, 1.55)	0.49
TZH * 2nd cosine function	1.74 (1.44, 2.09)	<0.0001	1.68 (1.41, 2.02)	<0.0001

[24] Some parameters are omitted because they were either to large to calculate or were excluded due to collinearity. BGD = Dhaka, Bangladesh; BRF = Fortaleza, Brazil; INV = Vellore, India; NEB = Bhaktapur, Nepal; PKN = Naushero Feroze Pakistan; PEL = Loreto, Peru; SAV = Venda, South Africa; TZH = Haydom, Tanzania.

Table S2: Adjusted effect – risk ratios, 95% confidence intervals (CI) and p-values from the final main effect and interaction Poisson models fitted with generalized linear models using deviations of the exposures from the site-specific average adjusting for seasonality. [24]

	Main effects model		Interaction model	
	**Risk ratio (95%CI)**	**p-value**	**Risk ratio**	**p-value**
**Precipitation deviations 8-day lag**		**<0.001**		**<0.0001**
Spline 1	1.08 (0.99, 1.18)	0.10	1.12 (1.02, 1.23)	0.02
Spline 2	0.08 (0.17, 0.43)	<0.01	0.04 (0.01, 0.21)	<0.001
Spline 3	1.46x10^{13} (5.92x10^{5}, 3.62x10^{20})	<0.001	3.68x10^{16} (1.33x10^{9}, 1.02x10^{24})	<0.0001
Spline 4	0.00 (0.00, 0.00)	<0.001	0.00 (0.00, 0.00)	<0.0001
**Relative humidity deviations 10-day lag**		**<0.0001**		**0.16**
Spline 1	1.01 (1.00, 1.02)	0.05	1.02 (0.96, 1.09)	0.56
Spline 2	0.96 (0.92, 1.00)	0.03	0.92 (0.72, 1.17)	0.51
Spline 3	1.59 (1.06, 2.39)	0.02	3.83 (0.37, 40.15)	0.26
Spline 4	0.31 (0.13, 0.75)	<0.01	0.21 (0.00, 3.13)	0.13
**Soil moisture 10-day lag**		**<0.001**		**<0.001**
Spline 1	0.97 (0.91, 1.04)	0.43	1.00 (0.94, 1.07)	0.95
Spline 2	0.72 (0.45, 1.15)	0.17	0.66 (0.40, 1.09)	0.11
Spline 3	20.38 (1.98, 210.05)	0.01	33.50 (1.71, 414.86)	0.01
Spline 4	0.01 (0.00, 0.13)	<0.01	0.00 (0.00, 0.08)	<0.01
**Solar radiation 2-day lag**		**0.01**		**0.21**
Spline 1	1.00 (1.00, 1.00)	0.75	1.06 (1.00, 1.12)	0.04
Spline 2	1.00 (0.98, 1.01)	0.81	0.77 (0.59, 1.00)	0.05
Spline 3	1.02 (0.95, 1.10)	0.59	2.79 (0.83, 9.30)	0.10
Spline 4	0.90 (0.78, 1.05)	0.192	0.19 (0.02, 1.96)	0.16
**Surface runoff 6-day lag**		**<0.0001**		**<0.01**
Spline 1	0.98 (2.05, 48.69)	<0.01	2.05x10^{5} (95.43, 4.38x10^{8})	<0.01
Spline 2	0.00 (0.00, 0.00)	<0.001	0.00 (0.00, 0.00)	<0.001
**Temperature 9-day lag**		**<0.0001**		**0.16**
Spline 1	0.97 (0.93, 1.00)	0.08	0.99 (0.85, 1.15)	0.93
Spline 2	1.23 (1.08, 1.40)	<0.01	1.22 (0.61, 2.46)	0.57
Spline 3	0.01 (0.00, 0.08)	<0.001	0.04 (0.00, 2.11x10^{5})	0.69
Spline 4	1.18x10^{4} (74.77, 1.88x10^{6})	<0.001	2.85x10^{3} (0.00, 6.26x10^{17})	0.64
**Wind speed 9-day lag**		**0.01**		**0.63**
Spline 1	0.87 (0.73, 1.04)	0.12	0.54 (0.16, 1.78)	0.31
Spline 2	0.96 (0.51, 1.82)	0.91	3.78 (0.08, 172.41)	0.50
Spline 3	2.38 (0.16, 34.85)	0.01	0.01 (0.00, 4.56x10^{4})	0.56
**Vaccine/age interaction**		-		**0.06**
Linear	-	-	0.90 (0.76, 1.07)	0.23
Quadratic	-	-	1.01 (0.99, 1.03)	0.25
Cubic	-	-	1.00 (1.00, 1.00)	0.35
**Vaccine/soil moisture interaction**		-		**<0.001**
Spline 1	-	-	0.56 (0.40, 0.80)	<0.01
Spline 2	-	-	24.90 (3.92, 158.01)	<0.01
Spline 3	-	-	0.00 (0.00, 0.00)	<0.001
Spline 4	-	-	1.14x10^{8} (7.38x10^{3}, 1.87x10^{12})	<0.001
**Vaccine/temperature interaction**		-		**<0.0001**
Spline 1	-	-	0.88 (0.73, 1.06)	0.19
Spline 2	-	-	0.74 (0.46, 1.20)	0.22
Spline 3	-	-	1.42x10^{4} (8.75, 2.30x10^{7})	0.01

Table S2: Adjusted effect – risk ratios, 95% confidence intervals (CI) and p-values from the final main effect and interaction Poisson models fitted with generalized linear models using deviations of the exposures from the site-specific average adjusting for seasonality. [24]

	Main effects model		Interaction model	
	**Risk ratio (95%CI)**	**p-value**	**Risk ratio**	**p-value**
Spline 4	-	-	0.00 (0.00, 0.05)	0.02
**Sample type/surface runoff interaction**		-		**<0.0001**
Spline 1	-	-	0.01 (0.00, 0.10)	<0.001
Spline 2	-	-	2.60×10^{9} (631.99, 1.07×10^{16})	<0.01
**Solar radiation/temperature interaction**		-		**<0.001**
Spline 1 * spline 1	-	-	1.00 (1.00, 1.00)	0.87
Spline 1 * spline 2	-	-	1.00 (1.00, 1.00)	0.91
Spline 1 * spline 3	-	-	0.98 (0.85, 1.13)	0.79
Spline 1 * spline 4	-	-	1.10 (0.79, 1.50)	0.60
Spline 2 * spline 1	-	-	1.00 (0.99, 1.01)	0.77
Spline 2 * spline 2	-	-	1.00 (0.97, 1.04)	0.93
Spline 2 * spline 3	-	-	1.03 (0.49, 2.17)	0.95
Spline 2 * spline 4	-	-	0.70 (0.14, 3.46)	0.67
Spline 3 * spline 1	-	-	0.98 (0.92, 1.05)	0.65
Spline 3 * spline 2	-	-	0.97 (0.80, 1.19)	0.79
Spline 3 * spline 3	-	-	1.94 (0.56, 67.3)	0.72
Spline 3 * spline 4	-	-	0.86 (0.00, 1.37×10^{3})	0.67
Spline 4 * spline 1	-	-	0.99 (0.81, 1.20)	0.89
Spline 4 * spline 2	-	-	1.11 (0.68, 1.81)	0.68
Spline 4 * spline 3	-	-	0.11 (0.00, 157.39)	0.55
Spline 4 * spline 4	-	-	13.54 (0.00, 2.03×10^{7})	0.72
**Solar radiation/surface runoff interaction**				**<0.01**
Spline 1 * spline 1	-	-	1.13 (1.00, 1.26)	0.04
Spline 1 * spline 2	-	-	0.46 (0.23, 0.91)	0.03
Spline 1 * spline 3	-	-	5.02×10^{13} (13.12, 1.92×10^{26})	0.03
Spline 1 * spline 4	-	-	0.00 (0.00, 0.06)	0.04
Spline 2 * spline 1	-	-	0.60 (0.35, 1.02)	0.06
Spline 2 * spline 2	-	-	30.94 (1.33, 719.52)	0.03
Spline 2 * spline 3	-	-	0.00 (0.00, 0.20)	0.05
Spline 2 * spline 4	-	-	3.71×10^{74} (0.29, 4.70×10^{149})	0.05
Spline 3 * spline 1	-	-	6.70 (0.61, 73.95)	0.12
Spline 3 * spline 2	-	-	0.00 (0.00, 2.13)	0.06
Spline 3 * spline 3	-	-	3.00×10^{227} (0.00, .)	0.08
Spline 3 * spline 4	-	-	0.00 (0.00, 3.43×10^{43})	0.09
Spline 4 * spline 1	-	-	0.08 (0.00, 6.69)	0.26
Spline 4 * spline 2	-	-	1.54×10^{9} (0.01, 2.80×10^{72})	0.10
**Relative humidity/temperature interaction**		-		**<0.001**
Spline 1 * spline 1	-	-	1.00 (1.00, 1.01)	0.31
Spline 1 * spline 2	-	-	0.99 (0.97, 1.01)	0.30
Spline 1 * spline 3	-	-	1.46 (0.86, 2.50)	0.16
Spline 1 * spline 4	-	-	0.42 (0.13, 1.39)	0.16
Spline 2 * spline 1	-	-	0.99 (0.97, 1.01)	0.27
Spline 2 * spline 2	-	-	1.06 (0.96, 1.16)	0.23
Spline 2 * spline 3	-	-	0.27 (0.04, 1.98)	0.20
Spline 2 * spline 4	-	-	13.22 (0.20, 895.99)	0.23
Spline 3 * spline 1	-	-	1.16 (0.93, 1.44)	0.18
Spline 3 * spline 2	-	-	0.57 (0.22, 1.44)	0.23
Spline 3 * spline 3	-	-	3.09×10^{4} (0.00, 3.61×10^{11})	0.40
Spline 3 * spline 4	-	-	0.00 (0.00, 1.05×10^{11})	0.50
Spline 4 * spline 1	-	-	0.72 (0.45, 1.15)	0.17
Spline 4 * spline 2	-	-	2.97 (0.41, 21.65)	0.28

Table S2: Adjusted effect – risk ratios, 95% confidence intervals (CI) and p-values from the final main effect and interaction Poisson models fitted with generalized linear models using deviations of the exposures from the site-specific average adjusting for seasonality. [24]

	Main effects model		Interaction model	
	**Risk ratio (95%CI)**	**p-value**	**Risk ratio**	**p-value**
Spline 4 * spline 3	-	-	0.00 (0.00, 1.43×10^{12})	0.76
Spline 4 * spline 4	-	-	2.15×10^{5} (0.00, 9.17×10^{39})	0.76
**Relative humidity/wind speed interaction**		-		**<0.001**
Spline 1 * spline 1	-	-	0.99 (0.92, 1.06)	0.73
Spline 1 * spline 2	-	-	1.03 (0.84, 1.27)	0.75
Spline 1 * spline 3	-	-	0.89 (0.40, 1.97)	0.78
Spline 2 * spline 1	-	-	1.08 (0.89, 1.31)	0.45
Spline 2 * spline 2	-	-	0.82 (0.43, 1.58)	0.56
Spline 2 * spline 3	-	-	1.87 (0.13, 26.54)	0.65
Spline 3 * spline 1	-	-	0.75 (0.14, 3.84)	0.73
Spline 3 * spline 2	-	-	2.03 (0.00, 836.00)	0.82
Spline 3 * spline 3	-	-	1.00 (0.00, 8.64×10^{10})	1.00
Spline 4 * spline 1	-	-	0.57 (0.02, 16.84)	0.75
Spline 4 * spline 2	-	-	7.88 (0.00, 3.35×10^{6})	0.76
Spline 4 * spline 3	-	-	0.00 (0.00, 3.84×10^{16}	0.55

Using earth observation-derived hydrometeorological variables to model and predict the probability of rotavirus infection in an eight-site cohort study, J. M. Colston et al. 2019, *The Lancet Planetary Health*

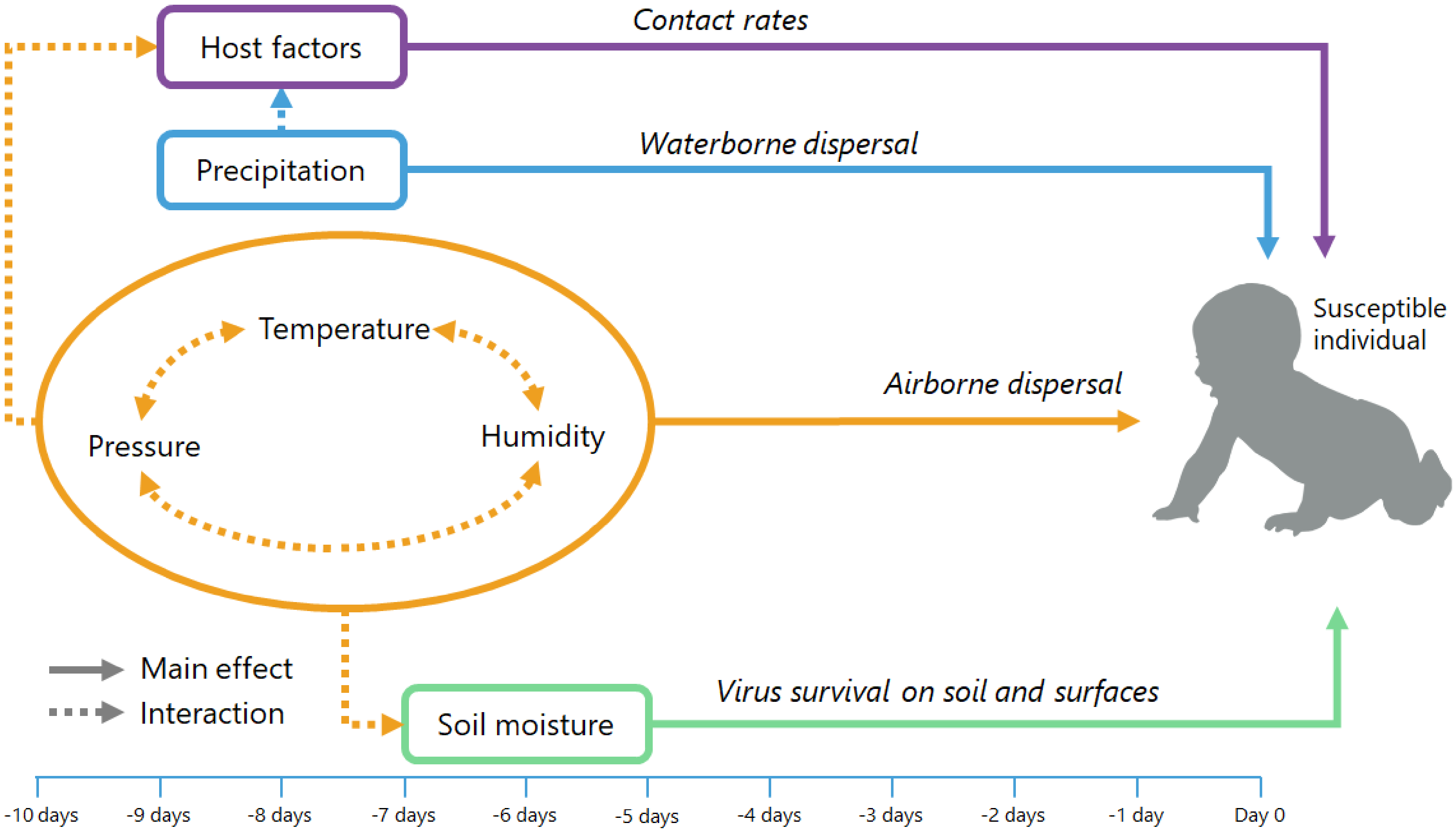

Figure S24: Hypothesized pathways by which hydrometeorological conditions influence risk of rotavirus transmission[25]

[25] Birth Clipart baby bundle 24, taken from https://clipart.wpblink.com/wallpaper-6355051

Using earth observation-derived hydrometeorological variables to model and predict the probability of rotavirus infection in an eight-site cohort study, J. M. Colston et al. 2019, *The Lancet Planetary Health*

Bibliography

Colston JM, Ahmed T, Mahopo C, Kang G, Kosek MN, de Sousa Junior F, et al. Evaluating meteorological data from weather stations, and from satellites and global models for a multi-site epidemiological study. Environ. Res. 2018;165:91–109.
